# Effects of Intermittent IL-2 Alone or with Peri-Cycle Antiretroviral Therapy in Early HIV Infection: The STALWART Study

**DOI:** 10.1371/journal.pone.0009334

**Published:** 2010-02-23

**Authors:** Jorge A. Tavel, INSIGHT STALWART Study Group

**Affiliations:** National Institute of Allergy and Infectious Diseases, National Institutes of Health, Bethesda, Maryland, United States of America; University of Sao Paulo, Brazil

## Abstract

**Background:**

The **St**udy of **Al**desleukin **w**ith and without **a**nti**r**etroviral **t**herapy (STALWART) evaluated whether intermittent interleukin-2 (IL-2) alone or with antiretroviral therapy (ART) around IL-2 cycles increased CD4^+^ counts compared to no therapy.

**Methodology:**

Participants not on continuous ART with ≥300 CD4^+^ cells/mm^3^ were randomized to: no treatment; IL-2 for 5 consecutive days every 8 weeks for 3 cycles; or the same IL-2 regimen with 10 days of ART administered around each IL-2 cycle. CD4^+^ counts, HIV RNA, and HIV progression events were collected monthly.

**Principal Findings:**

A total of 267 participants were randomized. At week 32, the mean CD4^+^ count was 134 cells greater in the IL-2 alone group (p<0.001), and 133 cells greater in the IL-2 plus ART group (p<0.001) compared to the no therapy group. Twelve participants in the IL-2 groups compared to 1 participant in the group assigned to no therapy experienced an opportunistic event or died (HR 5.84, CI: 0.59 to 43.57; p = 0.009).

**Conclusions:**

IL-2 alone or with peri-cycle HAART increases CD4^+^ counts but was associated with a greater number of opportunistic events or deaths compared to no therapy. These results call into question the immunoprotective significance of IL-2-induced CD4^+^ cells.

**Trial Registration:**

ClinicalTrials.gov NCT00110812

## Introduction

Antiretroviral therapy (ART) has led to significant suppression of HIV replication and improvement in both morbidity and mortality in patients with HIV-infection [1]–[3]. However, despite maximal viral suppression, viral eradication has not been achieved and viremia recurs in patients after treatment interruption [4]–[6]. Moreover, ART therapy is associated with significant toxicities, important interactions with other medications, difficulties in maintaining rigorous adherence, and its efficacy is limited by the emergence of drug resistant HIV variants [7]–[9]. Once ART has been started, lifelong therapy is required since treatment interruptions are associated with an increased risk of disease progression or death [10]. These limitations of ART have underscored the need to explore adjuvant or alternative therapeutic strategies for the treatment of HIV infection.

A number of randomized controlled trials have shown that the use of recombinant interleukin-2 (IL-2) with ART leads to significant and sustained increases in CD4^+^ counts in HIV-infected patients [11]–[22]. Although the use of IL-2 with antiretrovirals in the pre-ART era was associated with transient rises in plasma HIV RNA levels in some study participants, no clinical trial of IL-2 in HIV-infected patients has demonstrated a significant sustained increase in either plasma HIV RNA or intracellular HIV DNA in IL-2 recipients compared to controls. In fact, one randomized study actually showed a larger decrease in viral load after one year in participants given IL-2 and ART compared to those who received ART alone [22]. Similarly, a pooled analysis of long-term follow-up data from 3 randomized controlled trials showed that IL-2 used with combination ART produced significant decreases in viral load after a median of 30 months compared to ART alone [23]. While the success observed in increasing CD4^+^ counts without increasing viral load suggests IL-2 may be an effective strategy to complement the effects of ART in HIV-infected patients, it also raises the question of whether IL-2 can be used to maintain CD4^+^ counts while sparing the use of ART.

One small pilot trial, the United Kingdom Vanguard Study, showed that IL-2 used in the absence of concomitant ART boosted CD4^+^ counts in patients with early HIV disease and had no significant effect on viral load [24]. While that study provided promising data regarding the potential benefit and safety of IL-2 monotherapy, a relatively blunted CD4^+^ count response was observed when compared to results from three concurrent IL-2 studies that used IL-2 in combination with ART [16], [17], [23]. The average increase from baseline mean CD4^+^ count in the IL-2-treated group in the United Kingdom Vanguard Study was +129 compared to +13 cells/mm^3^ in the no IL-2 or ART group. In comparison, a meta-analysis showed the average increases from baseline in the IL-2 and ART-treated groups in the three other trials was +417 compared to +77 cells/mm^3^ in the ART alone groups [23], [24]. The reasons for this blunted CD4^+^ response to IL-2 are unknown, but one hypothesis is that ART combined with IL-2 has a synergistic effect to increase CD4^+^ count. This raises the possibility that antiretrovirals administered during the IL-2 cycle may yield a more robust immunologic response while sparing the adverse effects of continuous ART.

STALWART was designed to evaluate the safety and immunologic and virologic effects of intermittent IL-2 in asymptomatic, HIV-infected patients who did not meet criteria for initiation of ART. This study tested the hypothesis that intervention at an early stage of HIV infection with intermittent IL-2 therapy either alone or with peri-cycle ART would maintain or increase CD4^+^ counts compared to controls receiving neither ART nor IL-2. Concurrent with the STALWART study, two phase III trials were evaluating the clinical efficacy of intermittent IL-2 plus ART compared to ART alone in approximately 6,000 patients with HIV infection: ESPRIT (Evaluation of Subcutaneous Proleukin® in a Randomized International Trial) and SILCAAT (Subcutaneous Recombinant IL-2 in HIV-Infected Patients with Low CD4^+^ Counts under Active Antiretroviral Therapy). Both studies demonstrated that IL-2 in combination with continuous antiretrovirals did not result in a decreased incidence of a new or recurrent HIV disease progression event including death compared to ART alone [25], [26]. Based upon these results, IL-2 administration was halted in the STALWART study and safety and efficacy data were unblinded.

## Methods

### Study Design

The protocol for this trial and supporting CONSORT checklist are available as supporting information; see Checklist S1 and Protocol S1. STALWART was an international, multi-site, phase II open label randomized controlled study. Patients who met enrollment criteria were randomized in a 1∶1∶1 ratio to one of three groups: no therapy (control), IL-2 alone (IL-2), or IL-2 with peri-cycle ART (IL-2+ART). Randomization was performed using random permuted blocks stratified by individual clinical site. The random allocation sequence was generated centrally for each site. Sites accessed the randomization allocation sequence via the study website and the sequence was concealed until interventions were assigned. Dosing with IL-2 commenced at a dose of 7.5 million international units (MIU) administered by subcutaneous injection twice daily for a 5-day cycle. Dose reductions were permitted in 1.5 or 3.0 MIU/dose decrements. Both groups assigned to IL-2 were to receive a minimum of 3 IL-2 cycles separated by an interval of 8 weeks with additional cycles permitted at clinician discretion.

The choice of peri-cycle ART regimens in the IL-2+ART group was at the discretion of the site investigator, but regimens were required to consist of at least 1 protease inhibitor and 2 nucleoside or nucleotide reverse transcriptase inhibitors. Drug doses and schedules were determined according to treatment guidelines and package inserts. Because of possible hypersensitivity reactions with intermittent use of abacavir and drug resistance associated with intermittent use of NNRTIs, these drugs were excluded from the study. Participants in the IL-2+ART group commenced ART within 1 week of randomization and received an initial protocol-required 7–21 consecutive day course of antiretrovirals prior to the first IL-2 cycle. For subsequent cycles, ART was commenced 3 days prior to the initiation of IL-2 dosing, continued through the IL-2 cycle, and was stopped 2 days after IL-2 administration for a maximum of 10 days of ART around each cycle. Investigators could recommend that participants in any assigned group commence continuous ART at any time, and participants could independently decide to commence continuous ART for any reason. The primary outcome measure was the mean change in CD4^+^ count from baseline to week 32. Secondary outcome measures included grade 3 or 4 clinical events, mean change in plasma viral load from baseline to week 32, initiation of continuous antiretrovirals, and the development of HIV-1 drug resistance mutations in the IL-2+ART group.

The study was originally designed with a target sample size of 480 participants based on 80% power to detect a 50 to 75 cells/mm^3^ difference for each of the 3 planned pair-wise comparisons of the CD4^+^ count change. A 50 to 75 cell difference in CD4+ cell count was chosen because it was felt differences of that size would be clinically important to detect, and could influence how ART was used with IL-2. Due to a low rate of accrual and the anticipated closure of the two phase 3 IL-2 studies ESPRIT and SILCAAT, enrollment in STALWART was closed in June 2008. After ESPRIT and SILCAAT demonstrated that IL-2 in combination with continuous antiretrovirals did not result in a decreased incidence of a new or recurrent HIV disease progression event including death compared to antiretrovirals alone, IL-2 administration was halted in STALWART, and safety and efficacy data were unblinded. Follow-up continued through a common closing date of February 28, 2009 when all participants had completed their week 32 visit evaluations.

### Study Population

Eligible participants were at least 18 years of age, had documented HIV-infection, a CD4^+^ count ≥300 cells/mm^3^, and were antiretroviral naïve or had not received ART for at least one year prior to randomization. Exclusion criteria included significant renal, hepatic, gastrointestinal, central nervous system or hematologic disorders, or any history of autoimmune or inflammatory disease. Local Ethics Committee/Institutional Review Boards approved the study and written informed consent was obtained from all participants.

### Assessments

Study participants were required to have negative pregnancy tests and complete blood counts and serum chemistries within protocol-defined acceptable ranges, including hemoglobin levels above 10 gm/dL, granulocyte counts above 1000 cells/mm^3^ and AST and ALT below 5 times the upper limit of normal. All participants were seen at least monthly during the first 32 weeks of the study and at least every 4 months thereafter. Medical and drug histories were taken at each scheduled visit and CD4^+^ counts and HIV RNA, both performed locally by a laboratory participating in a certified quality assurance program, were measured. Complete blood counts and chemistries were performed at the beginning and during each IL-2 cycle. Participants receiving IL-2+ART had blood drawn during their third IL-2 cycle for HIV resistance genotyping (TruGene HIV-1 Genotyping Assay; Siemens Medical Solutions Diagnostics, Tarrytown, New York, USA). For those samples that demonstrated major antiretroviral resistance mutations as defined by the International Aids Society-USA Drug Resistance Mutations Group [27], stored plasma from their first cycle was sent for HIV genotyping to determine whether the mutations were present at baseline or had developed during the course of the study. Source documentation was provided by sites for opportunistic diseases and deaths that occurred during the study. Adverse clinical events (excluding opportunistic disease) were graded by site investigators using a standard NIAID Division of AIDS toxicity table. Grade 3 or 4 adverse events were reported for all 3 treatment groups for the duration of the trial, irrespective of perceived relationship to IL-2 or ART; these events were coded centrally using the Medical Dictionary for Regulatory Activities (MedDRA, version 12.0) by a nurse who was blinded to study treatment.

### Interim Safety and Efficacy Monitoring

An independent data and safety monitoring committee reviewed interim analyses of primary and secondary outcomes as well as safety at least annually.

### Statistical Analysis

Statistical analysis was performed using SAS software, version 9.1. Unless otherwise indicated, all analyses are intent-to-treat, and the p-values shown are two-sided.

The primary outcome, change in CD4^+^ count from baseline to Week 32, was analyzed using analysis of variance stratified by geographic region (7 strata total). All patients with data available at week 32 were included and baseline CD4+ cell count was included in the model as a covariate. An alpha level of .017 was pre-specified to control for type I error, and the 95% confidence intervals cited are adjusted for the 3 pairwise comparisons. All other analyses are presented unadjusted for multiple comparisons, using a nominal .05 level of significance. Stratified analysis of variance was also used to analyze HIV-RNA (log_10_ transformed). Time-to-event methods (stratified Cox proportional hazards models and Kaplan Meier survival curves) were used to analyze the incidence of adverse events and initiation of continuous ART. For participants who were event-free, follow-up time was censored at February 28, 2009 or on the date of loss to follow up, whichever occurred earlier. Hazard ratios are shown unadjusted.

## Results

### Study Population

**Figure 1:**
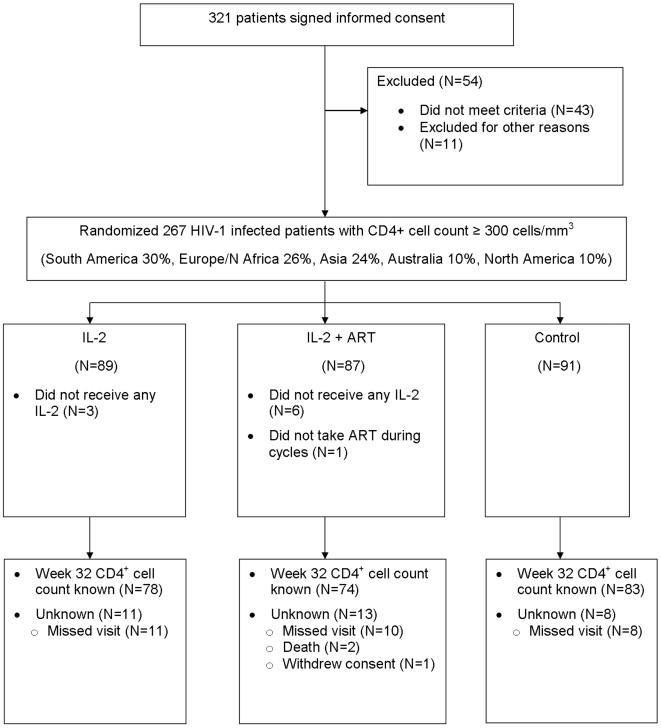
STALWART study design and CONSORT flow diagram.

From November 2005 to June 2008, 267 participants were randomized from 36 sites in 11 countries: 91 to the control group, 89 to IL-2, 87 to IL-2+ART (figure 1). The baseline median CD4^+^ count was 419 cells/mm^3^, and median HIV RNA was approximately 22,000 copies. Seventy-nine percent of participants were antiretroviral naïve. Baseline characteristics were similar across assigned treatment groups (table 1).

**Table 1:** Baseline characteristics by treatment group.

	IL-2	IL-2+ART	Control	Total
**Age (years)**				
Median	35	35	36	36
Interquartile range				30–44
**Gender (% female)**	19.1	14.9	17.6	17.2
**Race (%)**				
Asian	24.7	26.4	24.2	25.1
Black	4.5	4.6	8.8	6.0
White/other	70.8	69.0	67.0	68.9
**CD4+ count (cells/mm^3^)**				
Median	399	425	432	419
Interquartile range				359–512
**HIV-RNA (copies)**				
Median	23,508	21,881	24,328	22,384
Interquartile range				7,189–61,169
**Medication Use and History**				
Antiretroviral (ART) naïve (%)	76.4	87.4	72.5	78.7
**Months since last ART use** * **(median)**	36.9	28.9	32.0	21.3
Current OI prophylaxis use (%)	3.4	2.3	2.2	2.6
**Likely mode of HIV transmission** ** **(%)**				
Person of same sex	60.7	56.3	61.5	59.6
Person of opposite sex	38.2	41.4	37.4	39.0
Injection drug use	2.2	4.6	0.0	2.2
Blood products	1.1	1.1	0.0	0.7
Other or unknown	2.2	2.3	2.2	2.2
**Hepatitis Status (%)**				
Hepatitis B surface antigen positive	5.6	0.0	5.6	3.8
Hepatitis C antibody positive	5.7	5.7	7.8	6.4
**BMI (kg/m^2^)**				
Median	23.5	23.2	23.6	23.4
Interquartile range				21.2–26.2
**Total cholesterol (mg/dl)**				
Median	166	170	170	170
Interquartile range				147–196

*among antiretroviral experienced patients.

**more than one could be checked.

### Use of IL-2 and Peri-Cycle-ART

Sixty-seven (75%) and 55 (63%) of participants in the IL-2 and IL-2+ART groups received at least 3 cycles before 32 weeks. At the third cycle, the mean total dose was 64.0 vs. 63.3 MIU, respectively. The most commonly used peri-cycle ART regimens included a combination of ritonavir boosted lopinavir, atazanavir, or fosamprenavir with either zidovudine and lamivudine or tenofovir and emtricitabine.

### CD4+ Cell Count

**Figure 2:**
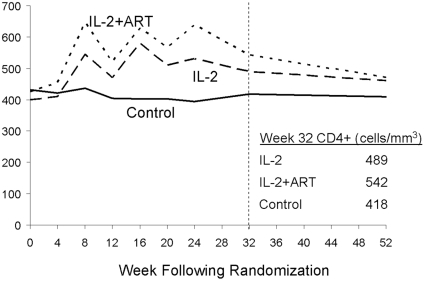
Median CD4+ cell count.

The primary outcome of week-32 CD4^+^ count was available for approximately 90% of living participants (figure 1). Figure 2 shows the median CD4^+^ count of the 3 groups at follow-up visits. At week 32, control participants had a mean change in CD4^+^ count of -22 cells/mm^3^ compared to +114 for those receiving IL-2 alone and +110 for those receiving IL-2+ART. Compared to the control participants, the mean change from baseline in CD4^+^ count (after adjustment for baseline CD4^+^ count) was higher by 134 cells/mm^3^ (95% CI: 70 to 198, p<0.001) in participants receiving IL-2 alone and higher by 133 cells/mm^3^ (95% CI: 68 to 199, p<0.001) in those receiving IL-2+ART. There was no difference between the two IL-2 groups (−0.5 cells/mm^3^; 95% CI: −67 to 66).

### HIV RNA Level

At week 32, participants receiving no IL-2 or ART had a mean change in log_10_ HIV RNA of −0.40 compared to −0.07 for those receiving IL-2 alone and −0.01 for those receiving IL-2+ART. Compared to the control participants, the mean change from baseline log_10_ viral load was +0.31 (95% CI: 0.08 to 0.55; p = 0.009) in participants receiving IL-2 alone and +0.37 (95% CI: 0.13 to 0.61; p<0.003) in those receiving IL-2+ART. The difference in HIV RNA between the two IL-2 groups was not statistically significant (0.05; 95% CI: −0.19 to 0.29; p = 0.67).

### Commencement of Continuous Antiretroviral Therapy

**Figure 3:**
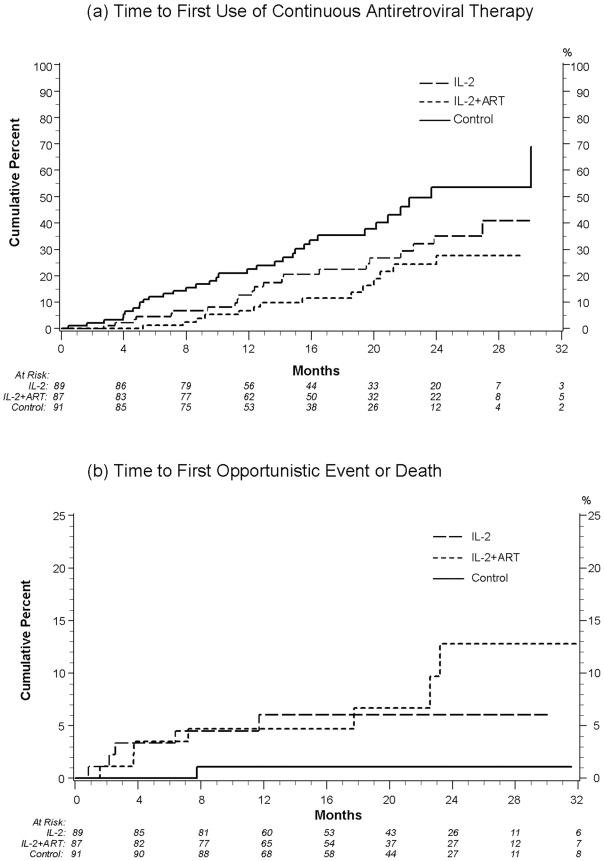
Time to first use of continuous antiretroviral therapy and time to first opportunistic event or death.

By the date of study closure, 34 participants assigned to no therapy had commenced continuous ART compared with 23 in the IL-2 alone and 14 in the IL-2+ART groups [figure 3(a)]. Using the control group as reference, the hazard ratio for commencing continuous ART was 0.58 (95% CI: 0.34 to 0.99; p = 0.048) for the IL-2 group and 0.32 (95% CI: 0.17 to 0.62; p<0.001) for the IL-2+ART group. The most common reason for starting continuous ART in all three groups was a low or declining CD4^+^ count.

### Moderate or Severe Adverse Events

Compared to the control group where eight participants experienced a moderate (grade 3) or severe (grade 4) event, 17 participants randomized to IL-2 alone [hazard ratio 2.60 (95% CI: 1.12 to 6.06; p = 0.03)] and 24 among those randomized to IL-2+ART [hazard ratio 4.05, 95% CI: 1.81 to 9.05; (p<0.001)] experienced at least one such event. As reported in previous studies of IL-2, common IL-2-related toxicities included fever, nausea, myalgias and rash. There was no statistically significant difference in the risk of a grade 3 or 4 event between participants receiving IL-2+ART versus those receiving IL-2 alone [hazard ratio 1.56; 95% CI: 0.83 to 2.91; (p = 0.17)].

### Genotyping Results

Fifty-four (62%) participants randomized to receive IL-2+ART completed 3 cycles of IL-2 and had specimens available for genotyping. Sequencing was successful in 47 participant samples. Two participants were found to have detectable HIV resistance mutations at cycle 3 (41L and 210W in one and 219Q in the other). The same mutations were found in samples saved at the end of cycle 1 from these patients, suggesting these TAMS were present at study entry, or developed during the first cycle of IL-2 administration.

### Clinical Outcomes

Table 2 shows opportunistic events and deaths by randomized group. The median follow-up was approximately 19 months. Five participants in the IL-2 alone group and 7 participants in the IL-2+ART group experienced an opportunistic event or died compared to one participant in the group assigned to no therapy. Compared to the control participants, the hazard ratio for developing an opportunistic disease or death from any cause was 5.08 (95% CI: 0.59 to 43.57; p = 0.14) for those receiving IL-2 alone and 6.56 (95% CI: 0.80 to 53.62; p = 0.08) for those receiving IL-2+ART. When the events in both IL-2 groups were pooled and compared to events in control participants, the hazard ratio was 5.84 (95% CI: 0.76 to 45.11; p = 0.09). [Figure 3(b).] The hazard ratio for clinical events excluding the two deaths was 4.88 (CI = 0.62 to 38.3, p = 0.13). No participant who experienced an event was on continuous ART when their event occurred.

**Table 2:** Reported disease progression events.

List of Reported Opportunistic Events					
Group	Event	No. cycles prior to event	Prox. CD4	Study Day Event Dx	Baseline CD4
IL-2	Toxoplasmosis	1	371	78	370
	PCP	1	390	66	310
	Tuberculosis	1	302	194	291
	Tuberculosis	3	148	356	298
	Bact. pneumonia initial	0	248	26	352
IL-2 + ART	Non-Hodgkins lymphoma	4	311	540	290
	Herpes zoster, multidermal	2	550	113	330
	Bact. pneumonia initial	3	474	706	451
	Bact. pneumonia initial	1	465	219	394
	Candidiasis esophageal	3	524	687	408
	Death, Motor Vehicle Accident	2	368	114	288
	Death Coronary Atherosclerosis	1	438	48	382
Control	Herpes zoster, multidermal	.	548	236	466

## Discussion

This is the largest clinical trial demonstrating that IL-2 alone in asymptomatic HIV-infected patients produces significant increases in CD4^+^ counts without changing plasma HIV RNA levels. Clinical trials of IL-2 administered with continuous antiretrovirals have demonstrated CD4^+^ count increases of 200 cells/mm^3^ or greater from baseline after 3 cycles. The magnitude of the CD4^+^ count increase from baseline seen after 3 cycles in this study – approximately 130 cells/mm^3^ at 32 weeks – was similar to that found in other studies that have evaluated IL-2 without continuous ART [24], [28]. This study was the first to evaluate the addition of peri-cycle ART to IL-2, and it was found that this strategy did not increase CD4^+^ cell response compared to IL-2 alone. There are at least 2 possible reasons why peri-cycle ART did not produce increases similar to continuous ART. First, the viral suppression afforded by peri-cycle ART may have been insufficient in duration or degree to create an appropriate immunologic milieu for a synergistic IL-2 response. Second, the greater increases in CD4^+^ count seen with IL-2 and continuous ART may not, in fact, be due to synergy between these drugs but instead reflect a simple additive effect of intermittent IL-2 and continuous ART-induced CD4^+^ cell increases.

The control group showed a significantly larger decrease in HIV RNA than either IL-2 group. When data were censored from participants who started who started continuous ART, there were no statistically significant changes in HIV RNA among the three groups. However, this *ad hoc* analysis is not protected by randomization and should be interpreted with caution.

Concurrent with the STALWART study, ESPRIT and SILCAAT were two clinical trials designed to evaluate the clinical efficacy of intermittent IL-2 plus continuous ART compared to ART alone in approximately 6,000 participants with HIV infection. Both studies demonstrated that, while IL-2 in combination with continuous ART did produce significant and sustained increases in CD4+ cell counts, this did not result in a decreased incidence of new or recurrent HIV disease progression events including death compared to ART alone. Of concern, IL-2 recipients in STALWART had a greater number of opportunistic diseases or deaths compared to controls that did not receive IL-2. The relationship of IL-2 to the events seen in STALWART must be interpreted cautiously. First, the overall number of events is small. Second, of the 12 events in IL-2 recipients, 5 occurred in participants who received only one cycle of IL-2, and another event occurred in a participant who was randomized to but never received IL-2. Nonetheless, the trend in clinical events observed in STALWART is concerning in light of the results of ESPRIT and SILCAAT that question the functionality of CD4^+^ cells induced by IL-2. Specifically, these results suggest that CD4^+^ counts may no longer serve as an accurate indicator of immune function in IL-2 recipients. In STALWART, the groups assigned to receive IL-2 started continuous ART less frequently than controls, and none of the participants with clinical events were on continuous ART prior to their event. Since a low CD4^+^ count was the most common reason given for starting continuous ART, one hypothesis to explain the greater number of clinical events in the IL-2 groups is that the IL-2-induced elevations in CD4^+^ counts, taken at face value, did not trigger the initiation of ART. Of note, another clinical trial comparing IL-2 monotherapy to no intervention, ANRS 119, also found that IL-2 recipients delayed ART initiation compared to controls, but an increase in clinical events was not observed: progression rates to AIDS or death were similar in the IL-2 (8%) and control (9%) arms [28].

IL-2 has been shown to raise CD4^+^ counts by expansion as well as increased survival of peripheral CD4^+^ cells [29], and it might be helpful to differentiate these IL-2 expanded – and possibly nonfunctional – cells in order to calculate a more accurate surrogate marker for disease progression in patients who have received IL-2. For example, the CD4^+^ cells expanded in the setting of IL-2 express increased levels of the alpha chain of the IL-2 receptor, CD25. Although this marker might be used to calculate a CD4^+^ count that more accurately represents an IL-2 recipient's immunological status, it is unlikely that a study will be undertaken to validate a new surrogate of immune status for this population. Therefore, the results from this trial have implications for the clinical management of patients who have received IL-2 since the CD4^+^ count may not accurately reflect the immunological status of these patients. First, in patients who have received IL-2 but who are not on continuous ART, antiretroviral treatment should be considered before these patients meet guideline-defined CD4^+^ criteria for commencing ART. Second, in those patients who have received IL-2 and are on continuous ART, careful and timely clinical evaluations should be undertaken if they present with symptoms of opportunistic diseases even if these occur in the setting of a high CD4^+^ count. If the IL-2 induced CD4+ count is no longer clinically useful as a surrogate of immune function and the duration of these CD4+ count increases is unknown, it would be of value to prolong the follow-up of STALWART participants. Therefore, in order to extend safety evaluations in the STALWART cohort, participants will be offered an additional two years of unblinded clinical follow up.

In summary, the STALWART study confirmed that IL-2 induces a significant increase in CD4^+^ count in HIV-infected patients not receiving continuous ART, and demonstrated that the magnitude of increase is not affected by the administration of short courses of peri-cycle ART. The CD4^+^ increases led to a significant delay in starting ART in IL-2 recipients, but this potential benefit appears to be offset by a potential increase in risk of clinical events. Taken together with the findings of ESPRIT and SILCAAT, this calls into question the ability of these IL-2 induced CD4+ cells to prevent opportunistic diseases.

### Writing Group

Jorge Tavel [chair], National Institute of Allergy and Infectious Diseases, Bethesda; Abdel Babiker, Medical Research Council, London; Lawrence Fox, National Institute of Allergy and Infectious Diseases, Bethesda; Daniela Gey, University of Copenhagen, Copenhagen; Gustavo Lopardo, FUNCEI, Buenos Aires; Norman Markowitz, Henry Ford Hospital, Detroit; Nicholas Paton, Medical Research Council, London; Deborah Wentworth, University of Minnesota, Minneapolis; Nicole Wyman, University of Minnesota, Minneapolis.

## Supporting Information

Protocol S1 Trial Protocol.
(0.44 MB PDF)

Checklist S1 CONSORT Checklist.
(0.19 MB DOC)
