# Supplementary material for: Effects of Intermittent IL-2 Alone or with Peri-Cycle Antiretroviral Therapy in Early HIV Infection: The STALWART Study
Source: PLoS One. 2010 Feb 23;5(2):e9334. doi: 10.1371/journal.pone.0009334 (PMC2826395; doi:10.1371/journal.pone.0009334)

# A Randomized, Open-Label, International Study of Subcutaneous Recombinant Interleukin-2 (rIL-2, Aldesleukin) with and without Concomitant Antiretroviral Therapy in Patients with HIV-1 Infection and CD4$^+$ Cell Counts $\geq$ 300/mm$^3$:

# Study of Aldesleukin with and without Antiretroviral Therapy (ESPRIT 002: STALWART)

**Sponsored by:**
National Institute of Allergy and Infectious Diseases (NIAID)

**Pharmaceutical Support Provided by:**
Chiron Corporation

**BB IND# 7250 Held by:**
Division of AIDS (DAIDS), NIAID

**Protocol Chair:** Jorge Tavel, M.D.
National Institute of Allergy and Infectious Diseases
9000 Rockville Pike, Building 10 Rm 11C103
Bethesda, MD 20892
(301) 402-0564
fax: (301)402-4097

## SYNOPSIS

**Purpose:**
The purpose of this study is to compare the effects of subcutaneous (SC) recombinant interleukin-2 (rIL-2) administered with and without concomitant pericycle highly active antiretroviral therapy (HAART) to no therapy on $CD4^+$ T lymphocyte count in patients with HIV-1 infection and $CD4^+$ T lymphocyte count ≥ 300 cells/$mm^3$.

**Design:**
International, phase II, multisite, open label, randomized, controlled trial

**Study Treatment:**
1. Eligible participants will be randomized in a 1:1:1 fashion to one of three groups:
   - Group A: no therapy
   - Group B: rIL-2 7.5 MIU SC b.i.d. for 5 consecutive days every 8 weeks for 3 cycles then as needed to maintain $CD4^+$ T cell counts at or above goal
   - Group C: rIL-2 7.5 MIU SC b.i.d. for 5 consecutive days every 8 weeks with HAART beginning 3 days prior to each cycle, continuing through the cycle, and stopping 2 days after the last day of rIL-2 administration (for a maximum of 10 days of HAART with each cycle) for 3 cycles then as needed to maintain $CD4^+$ T cell counts at or above goal
2. Participants randomized to groups B and C will receive cycles of rIL-2 unless toxicities or other contraindications develop.

**Sample Size:**
The total sample size is 480 participants with an equal allocation among the three study groups. Randomization will occur over a 12-month period. With this enrollment period and a proposed follow-up period to a common closing date 12 months after the last participant is randomized, the average follow-up will be 18 months.

**Inclusion Criteria:**
1. Documented HIV-1 infection by any licensed ELISA test and confirmed by a second method (e.g., Western Blot); or any one of the following prior to randomization: detectable HIV p24 antigen, quantifiable plasma HIV RNA, or proviral DNA
2. ≥ 18 years of age (children are not eligible for study participation because data on rIL-2 in pediatric HIV disease is limited, and more data on the effects of rIL-2 in the absence of antiretrovirals should be gathered in adults before exposing pediatric patients to these risks)
3. The following clinical laboratory values obtained within 45 days prior to randomization:
   a. One $CD4^+$ T cell count ≥ 300 cells/$mm^3$ (For participants who are status post-splenectomy, also a $CD4^+$ cell percentage on this occasion ≥ 20%.)
   b. AST or ALT < 5 X the upper limit of normal (ULN) range
   c. Total or direct bilirubin ≤ 2 X ULN (Participants with hyperbilirubinemia due to Gilbert's syndrome may have a serum bilirubin up to 5 X ULN)
   d. Serum creatinine ≤ 2 mg/dl (177 µmol/L)
   e. Sodium within normal limits
   f. Granulocyte count ≥ 1000/$mm^3$
   g. Hemoglobin ≥ 10 gm/dl
   h. Platelet count ≥ 50,000 cells/$mm^3$

4. Ability to provide informed consent
5. Ability to obtain HAART regimens consisting of ≥ 1 protease inhibitor and ≥ 2 nucleoside or nucleotide reverse transcriptase inhibitors

**Exclusion Criteria:**

1. Any prior history of rIL-2 use
2. Use of any approved or experimental antiretroviral drug (including hydroxyurea) within one year prior to randomization
3. In the judgment of the clinician, any current indication for continuous antiretroviral therapy, or any contraindication to antiretroviral therapy
4. Evidence of virological failure on a protease inhibitor- or nonnucleoside reverse transcriptase -based antiretroviral regimen
5. Use of systemic corticosteroids, chemotherapy, or experimental cytotoxic drugs within 45 days prior to randomization
6. Use of any agent (approved or experimental) with clinically significant immunomodulatory effects within 8 weeks prior to randomization
7. History of any AIDS-defining illness (category C., CDC, 1993) or any of the following conditions: extrapulmonary *Pneumocystis carinii* disease; multi-dermatomal *Herpes zoster* (≥ 10 lesions in a non-contiguous site); American trypanosomiasis (Chagas disease) of the CNS; *Penicillium marneffii* disease; visceral leishmaniasis; non-Hodgkin's lymphoma of any cell-type; Hodgkin's lymphoma; bartonellosis; microsporidiosis (> 1 month's duration); nocardiosis; invasive aspergillosis; or *Rhodococcus equi* disease
8. Concurrent malignancy requiring cytotoxic chemotherapy
9. Any CNS abnormality that requires ongoing treatment with antiseizure medication
10. Current or historical autoimmune/inflammatory diseases including:
    a. Inflammatory bowel disease
    b. Psoriasis
    c. Optic neuritis
    d. Any autoimmune/inflammatory diseases with potentially life-threatening complications
11. Significant cardiac, pulmonary, renal, hepatic, gastrointestinal, CNS, psychiatric disease or illicit substance use/abuse that in the opinion of the investigator would make the participant a poor candidate for study participation
12. Pregnancy (for women of childbearing potential, a negative pregnancy test, urine or serum, is required within 14 days prior to randomization)
13. Breastfeeding

**Primary Outcome Measure:**

The primary outcome measure will be mean change in $CD4^+$ T lymphocyte count from baseline (average of two pre-randomization counts) to Week 32 in the three study groups.

**Secondary Outcome Measures:**

1. Grade 3 and 4 events
2. Therapy modification defined as any of the following:
    a. Permanent discontinuation of rIL-2 in groups B and C
    b. Changes in antiretroviral regimen in group C due to toxicity
    c. Initiation of continuous antiretroviral therapy in groups A, B and C
3. Plasma HIV RNA (evaluated at week 32 and month 12)

4. $CD4^+$ T lymphocyte (evaluated at month 12)
5. HIV-1 genotype changes
6. Fasting lipid profile, thyroid stimulating hormone and hepatic transaminase levels

**I. INTRODUCTION** ..... **1**

**II. PURPOSE** ..... **4**

**III. STUDY OBJECTIVES** ..... **4**

**IV. STUDY OUTCOME MEASURES** ..... **4**

**V. STUDY DESIGN** ..... **5**

**VI. PARTICIPANT POPULATION** ..... **5**
**A. Inclusion Criteria** ..... **5**
**B. Exclusion criteria** ..... **6**

**VII. TREATMENT PROCEDURES** ..... **7**
**A. Product Descriptions** ..... **7**
1. Recombinant rIL-2 ..... 7
2. Antiretrovirals ..... 7
**B. Product Dosing and Schedules** ..... **7**
1. rIL-2 in Groups B and C ..... 7
2. Antiretrovirals in Group C ..... 7
**C. rIL-2 Guidelines and Dose Modification** ..... **8**
1. rIL-2 dose modifications for management of toxicities ..... 8
2. rIL-2 schedule modifications based on virologic monitoring ..... 9
3. rIL-2 schedule modifications based upon immunologic monitoring ..... 10
**D. Antiretroviral guidelines and dose modification** ..... **10**
1. Antiretroviral dose modifications for management of toxicities ..... 11
2. Antiretroviral modifications in response to rIL-2 modifications ..... 11
**E. Concomitant Therapy** ..... **11**
1. Antiretroviral therapy ..... 11
2. Antimicrobial prophylaxis ..... 12
3. Treatment of intercurrent illnesses ..... 12
4. Symptomatic management during rIL-2 treatments ..... 12

**VIII. STUDY PROCEDURES** ..... **12**
**A. General** ..... **12**
**B. Baseline Screening and Enrollment** ..... **13**
**C. Study evaluations** ..... **13**
1. Group A Participants (no therapy) ..... 14
2. Group B and C Participants (rIL-2 recipients) ..... 14
3. HIV Genotyping ..... 14
**D. Follow-up Evaluations (after Week 32)** ..... **15**
1. 4-Month Follow-up Visits (All Groups) ..... 15
2. rIL-2 Cycles (rIL-2 Groups Only) ..... 15
**E. Stored Samples and Future Research** ..... **16**

**IX. STUDY TREATMENT DISCONTINUATION ........ 16**

**X. STATISTICAL CONSIDERATIONS ........ 16**
**A. Sample Size ........ 16**
**B. Data Collection and Analyses ........ 17**

**XI. HAZARDS/DISCOMFORTS/RISKS ........ 18**
**A. Treatments ........ 18**
1. rIL-2 ........ 19
2. Antiretrovirals ........ 19
**B. Procedures ........ 19**
**C. Stored Samples, Medical Records and Confidentiality ........ 20**

**XII. EVENT REPORTING ........ 20**
**A. Disease Progression Events (All treatment groups) ........ 20**
**B. Grade 3 and 4 Events and Death (All treatment groups) ........ 20**
**C. Expedited Adverse Experiences (rIL-2 Groups Only) ........ 20**
1. EAE Reporting to DAIDS ........ 21
2. EAE Reporting Requirements ........ 21
3. STALWART EAE Definition ........ 22

**XIII. MONITORING ........ 22**

**XIV. BENEFITS ........ 22**

**XV. COMPENSATION ........ 22**

**XVI. FINANCIAL DISCLOSURE ........ 22**

**XVII. REFERENCES ........ 24**

**APPENDIX A SAMPLE INFORMED CONSENT**
**APPENDIX B TOXICITY TABLE FOR GRADING ADVERSE EVENTS**
**APPENDIX C GUIDELINES FOR IL-2 DOSE MODIFICATIONS**
**APPENDIX D AIDS-DEFINING ILLNESSES AND OTHER CONDITIONS CONSIDERED EVIDENCE OF DISEASE PROGRESSION**
**APPENDIX E TIME AND EVENTS SCHEDULE**

# I. INTRODUCTION

Highly active antiretroviral therapy (HAART) has lead to significant suppression of HIV replication and improvement in both morbidity and mortality in HIV-infected patients [1-3]. However, despite maximal viral suppression, viral eradication has not been achieved and viremia typically recurs in patients after treatment interruption [4-6]. Moreover, HAART therapy is associated with significant toxicities [7], difficulties in maintaining rigorous compliance [8], and its efficacy is limited by drug resistant HIV variants [9]. These limitations of HAART have underscored the need to explore adjuvant therapies or alternative treatment strategies for the treatment of HIV infection. Considerable efforts have been made to complement HAART with manipulation of the immune system in order to improve overall clinical outcome. $CD4^+$ T lymphocyte count is known to be an important prognostic factor for clinical outcome in HIV infection [10]. Consequently, one investigational strategy has been to use various immune-based strategies to replenish the $CD4^+$ T cell pool and preserve or even augment immune function.

Recombinant interleukin 2 (rIL-2), originally described as T-cell growth factor and currently licensed in the United States for use in patients with metastatic melanoma and renal cell carcinoma, is one agent that has been extensively evaluated in patients with HIV infection since the early years of the AIDS epidemic [11]. A number of randomized controlled trials have shown that the use of rIL-2 with combination antiretroviral therapy leads to substantial increases in $CD4^+$ T lymphocyte counts in HIV-infected patients [12-22]. These increases in $CD4^+$ T cell count have been demonstrated to persist for years after rIL-2 administration [23]. The use of rIL-2 with antiretrovirals in the pre-HAART era was associated with transient rises in plasma HIV RNA levels in some participants. But no clinical trial of rIL-2 in HIV-infected participants has demonstrated a significant sustained increase in viral load in rIL-2 recipients compared to controls. CPCRA 059 was the largest phase II trial designed to investigate the effect of rIL-2 on plasma HIV RNA over one year. In this 511 patient randomized study, rIL-2 recipients exhibited no difference compared to controls in percentage of patients with viral loads <50 copies/ml, time to viral load ≥50 copies/ml for patients who had baseline viral loads <50 copies/ml, and change in viral load from baseline in patients with viral load >50 copies/ml at baseline [22]. One randomized study actually showed a larger decrease in viral load after one year in patients on rIL-2 and antiretroviral therapy compared to antiretroviral therapy alone [20]. Similarly, a pooled analysis of long-term follow-up data from 3 randomized controlled trials showed that rIL-2 used with combination antiretroviral therapy produced significant decreases in viral load after a median of 30 months compared to antiretroviral therapy alone [24]. While the success observed in increasing $CD4^+$ T lymphocyte counts without increasing viral load suggests rIL-2 may be an effective strategy to complement the effects of antiretrovirals in HIV-infected patients, it also raises the question of whether rIL-2 can be used to maintain $CD4^+$ T cell counts while sparing the use of HAART.

Early studies of intravenous rIL-2 in the pre-HAART era enrolled participants receiving what is now considered substandard antiretroviral therapy. In one randomized study [12], 30 participants who received rIL-2 had mean viral loads of approximately 40,000 copies/ml. Despite this viremia, these 30 participants demonstrated significant increases in $CD4^+$ T lymphocyte counts without sustained increases in HIV load compared to the 30 participants randomized to receive antiretroviral therapy alone. Therefore, neither does rIL-2 adversely

affect HIV load in participants receiving substandard antiretroviral therapy, nor does substandard antiretroviral therapy eliminate rIL-2-induced improvements in $CD4^+$ lymphocyte counts.

A small pilot trial, the United Kingdom Vanguard Study, showed that rIL-2 used in the absence of concomitant antiretrovirals boosts $CD4^+$ T cell counts in patients with early HIV disease while having no statistically significant effect on viral load [25]. In this study, 36 antiretroviral naïve HIV-infected patients with a baseline $CD4^+$ T lymphocyte count ≥ 350 cells/$mm^3$ were randomly assigned to receive either 3 5-day cycles of rIL-2 alone or no treatment. rIL-2 administered in the absence of concomitant antiretrovirals was well tolerated and resulted in a significant increase from baseline in time-weighted mean $CD4^+$ T lymphocyte count compared to controls (107 vs. 11 cells/ $mm^3$; p=0.002; Figure 1). Despite a transient increase in plasma HIV RNA levels at the end of the 5-day rIL-2 cycle, there was no significant or sustained increase in viral load over the 24-week follow-up period in those randomized who received rIL-2 compared to controls (Table 1).

**Figure 1**. United Kingdom Vanguard Study mean change from baseline $CD4^+$ T lymphocyte count with 95% confidence intervals

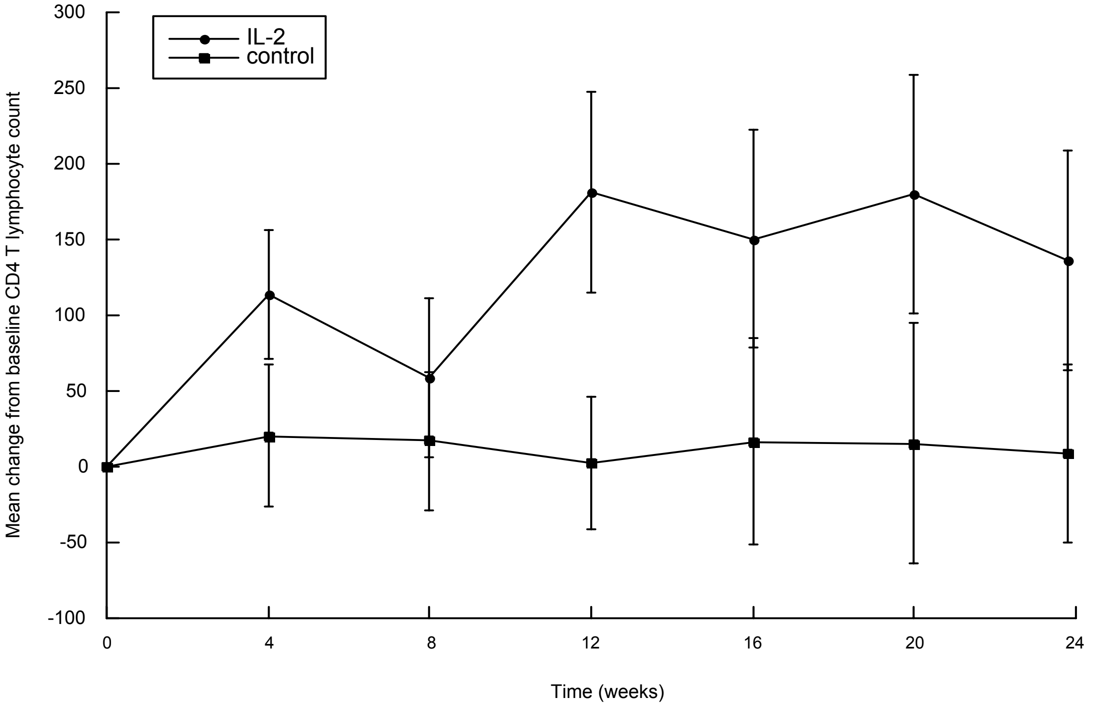


| | | | | | | | |
|---|---|---|---|---|---|---|---|
| IL-2 | 24 | 24 | 24 | 24 | 24 | 24 | 24 |
| Control | 12 | 12 | 12 | 12 | 12 | 11 | 11 |

**Table 1.** United Kingdom Vanguard Study mean change from baseline plasma HIV RNA.

| | rIL-2 Group | | Control Group | | |
|---|---|---|---|---|---|
| Time (weeks) | Mean change Log 10 copies per milliliter | Standard error | Mean change Log 10 copies per milliliter | Standard error | P-value* |
| Week 8 | +0.21 | 0.11 | +0.12 | 0.11 | 0.29 |
| Week 16 | +0.15 | 0.09 | +0.03 | 0.09 | 0.19 |
| Week 24 | +0.18 | 0.09 | +0.22 | 0.06 | 0.97 |

* Adjusted for baseline HIV RNA

While this study provided promising data regarding the use of rIL-2 monotherapy in asymptomatic, antiretroviral-naive HIV-infected patients, participants showed a relatively blunted CD4+ T lymphocyte count response compared to participants in three concurrent rIL-2 Vanguard Studies (Argentina, Thailand and Houston, Texas) that used rIL-2 in combination with antiretrovirals. The average increase in the time-weighted mean CD4+ T cell count in the rIL-2-treated group in the United Kingdom Vanguard Study was 107 cells/mm$^3$ from baseline, while the average increases from baseline in rIL-2-treated groups in the other Vanguard Studies were 328, 459, and 347 cells/ mm$^3$ [16, 17]. The reasons for this are unknown, but one possible explanation is that antiretrovirals produce a synergistic effect with rIL-2 in increasing CD4$^+$ T cell count. This raises the possibility that antiretrovirals administered during the rIL-2 cycle may yield a more robust immunologic response while sparing the adverse effects of continuous HAART use.

Another small pilot study, ICARUS (Interrupted versus Continuous Antiretrovirals involving Randomization from the Umbrella Study), randomized 39 HIV infected patients with prior HAART and rIL-2 exposure, CD4$^+$ T cell counts of at least 500 cells/mm$^3$ and any plasma HIV load to receive either continuous HAART in combination with intermittent subcutaneous rIL-2 (control group) or rIL-2 with pericycle HAART (interruption group). In the interruption group, HAART was administered for 3 days prior, 5 days during, and 2 days after rIL-2 cycles. As of September 2004, 20 rIL-2 cycles with pericycle HAART had been administered to 15 patients in the interruption group. Compared to the continuous group, the intermittent group had similar rates of moderate and severe toxicities. Since viral genotyping has not yet been performed in this study, it is unknown whether pericycle HAART led to an increased rate of resistance compared to continuous HAART.

Two ongoing phase III trials are studying the clinical efficacy of intermittent rIL-2 plus antiretrovirals compared to antiretrovirals alone in patients with HIV infection: ESPRIT (Evaluation of Subcutaneous Proleukin® in a Randomized International Trial) and SILCAAT (Subcutaneous recombinant rIL-2 in HIV infected patients with low CD4 Counts under Active Antiretroviral Therapy). ESPRIT is a 4000 patient clinical trial of patients with CD4$^+$ T lymphocyte counts ≥300 cells/mm$^3$. SILCAAT is a 2000 patient clinical trial studying rIL-2 in patients with CD4$^+$ T lymphocyte counts between 50 and 299 cells/mm$^3$. The primary endpoint

of both studies is time to a new or recurrent disease progression event including death. If rIL-2 is shown to be clinically effective in either or both of these studies, rIL-2 could serve an important role as an antiretroviral-sparing agent.

STALWART is designed to evaluate the safety and immunologic and virologic effects of intermittent rIL-2 in asymptomatic, HIV-infected patients who do not meet criteria for initiation of HAART. The hypothesis being tested is that intervention at an early stage of HIV infection with intermittent rIL-2 therapy either alone or with pericycle antiretroviral therapy can maintain or increase $CD4^+$ T cell counts as compared to controls who receive neither antiretroviral therapy nor rIL-2. Subsequent studies could then address whether these rIL-2-induced effects on $CD4^+$ T lymphocyte count delay commencement or reduce the use of antiretroviral medications.

## II. PURPOSE

The purpose of this study is to compare the effects of intermittent cycles of subcutaneous rIL-2 administered with and without concomitant pericycle highly active antiretroviral therapy to no therapy on $CD4^+$ T lymphocyte count in patients with HIV-1 infection and $CD4^+$ T lymphocyte count ≥ 300 cells/mm$^3$.

## III. STUDY OBJECTIVES

### A. Primary Objective

To compare the change from baseline in $CD4^+$ T lymphocyte count after 32 weeks in groups of participants randomly assigned to receive no therapy (no antiretrovirals or rIL-2), subcutaneous rIL-2 monotherapy or subcutaneous rIL-2 with pericycle HAART.

### B. Secondary Objectives

To compare among the three treatment groups after 32 weeks:

1. incidence of grade 3 and 4 events
2. number of therapy modifications
3. mean plasma HIV RNA changes from baseline (evaluated at 32 weeks and 12 months)
4. mean $CD4^+$ T lymphocyte changes from baseline at 12 months
5. changes in HIV genotyping that may represent development of antiretroviral drug resistance
6. selected lipid, thyroid, and hepatic abnormalities

## IV. STUDY OUTCOME MEASURES

### A. Primary Outcome Measure

The primary endpoint will be mean change in $CD4^+$ T lymphocyte count from baseline (average of two pre-randomization counts) to 32 weeks in the three study groups.

### B. Secondary Outcome Measures

1. Grade 3 and 4 events
2. Therapy modification defined as any of the following:
   a. permanent discontinuation of rIL-2 in groups B and C

   b. changes in antiretroviral regimen in group C due to toxicity
   c. initiation of continuous antiretroviral therapy in groups A, B and C
3. Plasma HIV RNA (evaluated at 32 weeks and 12 months)
4. $CD4^+$ T lymphocytes evaluated at 12 months
5. HIV-1 genotype changes
6. Fasting lipid profile, thyroid stimulating hormone and hepatic transaminase levels

## V. STUDY DESIGN

This is an international, phase II multi-site, open-label, randomized controlled study of subcutaneous rIL-2 alone vs. rIL-2 plus HAART administered only during rIL-2 cycles vs. no therapy in asymptomatic, HIV-infected patients with a $CD4^+$ T cell count of at least 300 cells/mm$^3$. There will be three treatment arms. Eligible patients who provide consent will be randomized as per Figure 2. Participants will be followed until a common closing date which will be 12 months after the last participant is randomized.

**Figure 2. Study Randomization**

Randomization (1:1:1) within strata defined by clinical site:

**N=480**

- **Group A** N=160; No therapy
- **Group B** N=160, rIL-2 (7.5 MIU sc q12 hours x 5 days q8 weeks x 3 cycles, then q8 weeks as needed to maintain counts at or above goal)
- **Group C** N=160; rIL-2 (7.5 MIU sc q12 hours x 5 days q8 weeks x 3 cycles, then q8 weeks as needed to maintain counts at or above goal) + HAART (3 days prior to, 5 days during, and 2 days after rIL-2 cycle)

## VI. PARTICIPANT POPULATION

### A. Inclusion Criteria

Potential participants must fulfill all of the following inclusion criteria to be eligible for randomization:

1. Documented HIV-1 infection by any licensed ELISA test and confirmed by a second method (e.g., Western Blot); or any one of the following prior to randomization: detectable HIV p24 antigen, quantifiable plasma HIV RNA, or proviral DNA.
2. ≥ 18 years of age (children are not eligible for study participation because data on rIL-2 in pediatric HIV disease is limited, and more data on the effects of rIL-2 in the absence of antiretrovirals should be gathered in adults before exposing pediatric patients to these risks).

3. The following clinical laboratories obtained within 45 days before randomization:
   a. One $CD4^+$ T cell count ≥ 300 cells/$mm^3$ (For participants who are status post-splenectomy, also a $CD4^+$ cell percentage on this occasion ≥ 20%.)
   b. AST or ALT < 5 X the upper limit of normal (ULN) range
   c. Total or direct bilirubin ≤ 2 X ULN (Participants with hyperbilirubinemia due to Gilbert's syndrome may have a serum bilirubin up to 5 X ULN.)
   d. Serum creatinine ≤ 2 mg/dl (177 µmol/L)
   e. Sodium within normal limits
   f. Granulocyte count ≥ 1000/$mm^3$
   g. Hemoglobin ≥ 10 gm/dl
   h. Platelet count ≥ 50,000 cells/$mm^3$
4. Ability to provide informed consent
5. Ability to obtain HAART regimens consisting of ≥ 1 protease inhibitor and ≥ 2 nucleoside or nucleotide reverse transcriptase inhibitors

**B. Exclusion criteria**

1. Any prior history of rIL-2 use
2. Use of any approved or experimental antiretroviral drug (including hydroxyurea) within one year prior to randomization
3. In the judgment of the clinician, any current indication for continuous antiretroviral therapy, or any contraindication to antiretroviral therapy
4. Evidence of virological failure on a protease inhibitor or nonnucleoside reverse transcriptase - based antiretroviral regimen
5. Use of systemic corticosteroids, chemotherapy, or experimental cytotoxic drugs within 45 days prior to randomization
6. Use of any agent (approved or experimental) with clinically significant immunomodulatory effects within 8 weeks prior to randomization
7. History of any AIDS-defining illness (category C., CDC, 1993) or any of the following conditions: extrapulmonary *Pneumocystis carinii* disease; multi-dermatomal *Herpes zoster* (≥ 10 lesions in a non-contiguous site); American trypanosomiasis (Chagas disease) of the CNS; *Penicillium marneffii* disease; visceral leishmaniasis; non-Hodgkin's lymphoma of any cell-type; Hodgkin's lymphoma; bartonellosis; microsporidiosis (> 1 month's duration); nocardiosis; invasive aspergillosis; or *Rhodococcus equi* disease
8. Concurrent malignancy requiring cytotoxic chemotherapy
9. Any CNS abnormality that requires ongoing treatment with antiseizure medication
10. Current or historical autoimmune/inflammatory diseases including:
    a. Inflammatory bowel disease
    b. Psoriasis
    c. Optic neuritis
    d. Any autoimmune/inflammatory diseases with potentially life-threatening complications
11. Significant cardiac, pulmonary, renal, hepatic, gastrointestinal, CNS, or psychiatric disease or illicit substance use/abuse that in the opinion of the investigator would make the participant a poor candidate for study participation

12. Pregnancy (for women of childbearing potential, a negative pregnancy test, urine or serum, is required within 14 days prior to randomization)
13. Breastfeeding

## VII. TREATMENT PROCEDURES

### A. Product Descriptions

1. Recombinant rIL-2

Chiron's recombinant interleukin-2 (rIL-2, aldesleukin) is provided in two vial sizes, 1.3 mg [18 million International Units (MIU)] and 0.44 mg (4.5 MIU). It is supplied as a sterile, white to offwhite, lyophilized cake in single-use vials. For the 1.3 mg ("18 MIU") vial, when reconstituted with 1.2 mL Sterile Water for Injection (SWFI), each mL contains 18 MIU (1.1 mg) rIL-2. For the 0.44 mg ("4.5 MIU") vial, when reconstituted with 0.4 mL SWFI, each 0.25 mL contains 4.5 MIU rIL-2. Vials of lyophilized rIL-2 must be stored at 2° to 8°C. Please refer to the *Chiron Investigator's Brochure for Aldesleukin (Proleunkin ) Recombinant Human Interleukin-2 (IL-2) for Use in HIV disease* [26] for complete formulation and reconstitution information. Common toxicities of rIL-2 are listed in Section XI.

2. Antiretrovirals

Information on formulation, toxicities, dosing, stability, and storage of antiretroviral drugs can be found in the package inserts for licensed therapies as provided.

### B. Product Dosing and Schedules

1. rIL-2 in Groups B and C

Dosing with rIL-2 should commence at a dose of 7.5 MIU administered by subcutaneous injection twice daily for 5 days (a day is defined as a 24 hour period) every 8 weeks (± 2 weeks). Time between cycles is counted from the first day of one cycle to the first day of the next. Dosing will not extend beyond a 5-day period regardless of missed doses or dose reductions.

2. Antiretrovirals in Group C

HAART regimens must consist of ≥ 1 protease inhibitor and ≥ 2 nucleoside or nucleotide reverse transcriptase inhibitors dosed according to current treatment guidelines and package inserts. The choice of antiretroviral regimen will be at the discretion of the investigators at each study site. Abacavir will not be permitted as pericycle antiretroviral therapy because of the potential for hypersensitivity reactions with intermittent abacavir therapy. Nonnucleoside reverse transcriptase inhibitors (NNRTIs) will not be permitted as pericycle antiretroviral therapy because of the potential for resistance with intermittent NNRTI therapy. Participants with active hepatitis B infections (i.e., hepatitis B surface antigen positive) should not receive intermittent lamivudine, tenofovir or emtricitabine because of the potential to develop resistant hepatitis B.

Group C participants should commence HAART within 1 week of randomization, and will receive 14 consecutive days of antiretrovirals (±7 days, for a range of 1 to 3 weeks on HAART) prior to the first rIL-2 cycle. If an intercurrent illness occurs after randomization that could delay the first rIL-2 cycle, the clinician may choose to delay HAART until the illness has resolved. HAART administration will then continue through the first rIL-2 cycle, and stop 2 days after rIL-2 is completed. For subsequent cycles, HAART will commence 3 days prior to the initiation of rIL-2 dosing, continue through the rIL-2 dosing, and stop 2 days after rIL-2 administration is completed, for a maximum of 10 days of HAART around each cycle.

Antiretrovirals in Group C will be administered only in conjunction with rIL-2 cycles. It is advised that a regimen with a minimal side effect profile be chosen since antiretroviral discontinuation due to intolerance will automatically lead to premature rIL-2 cycle termination in Group C.

**C. rIL-2 Guidelines and Dose Modification**

1. rIL-2 dose modifications for management of toxicities

Participants should be monitored closely for side effects of rIL-2. Participants who experience dose-limiting side effects may have their rIL-2 temporarily interrupted or their dose reduced. Appendix C includes a list of toxicities for which the investigator may choose to temporarily interrupt or dose-reduce rIL-2. All dose reductions must be in 1.5 or 3.0 MIU/dose decrements. The minimum dose of rIL-2 that can be delivered is 1.5 MIU b.i.d. The maximum dose is 7.5 MIU b.i.d. The maximum duration of each cycle of dosing will be 5 consecutive days and will not be extended for doses missed due to management of toxicities.

Recommended guidelines for management of toxicities that occur during cycles of rIL-2 are as follows:

- For toxicities during the 5-consecutive-day administration of rIL2 that are considered dose-limiting, the remaining rIL-2 doses of that cycle of therapy should be interrupted until the dose-limiting toxicity is no longer considered dose-limiting.

- Upon resolution of the dose-limiting toxicity, if the clinician judges the toxicity to be no longer dose-limiting, the participant may resume dosing with rIL-2 at the same dose or a reduced dose for the remainder of the 5-consecutive-day cycle in which the dose-limiting toxicity occurred. Dosing will continue to be delivered on a q12h schedule. Participants may have their dose reduced twice during a cycle. Participants who require more than two dose modifications in a cycle of therapy should have that cycle of treatment terminated.

- Participants who require dose modifications during a cycle of therapy are encouraged to have the next cycle initiated at a dose below the level that gave rise to the dose-limiting toxicity during the previous cycle. Subsequent dose reductions should occur, as necessary, in decrements of 1.5 or 3.0 MIU/dose delivered on a q12h schedule.

- If a participant who was previously dose-reduced completes a subsequent 5-consecutive-day treatment cycle at the reduced dose, without toxicity, then escalation of rIL-2 dose at the beginning of the next cycle may occur, by an increment of 1.5 or 3.0 MIU/dose.

Toxicities which lead to dose modifications will be recorded on data collection forms. In addition, documentation of all relevant clinical evaluations should be included in the participant's clinical/medical record in accordance with standard procedures.

Certain events or conditions may require delaying the next rIL-2 treatment cycle or permanently discontinuing rIL-2. Participants who experience any of the following events or conditions should have their rIL-2 cycle delayed:

- Pregnancy or breastfeeding
- Intercurrent illness that, in the judgment of the clinician, would significantly affect assessment of clinical status
- Use of cytotoxic agents for any reason
- Toxicity requiring temporary discontinuation

Women who become pregnant will remain in follow-up and can re-start treatment with rIL-2 after the pregnancy is over and after they have stopped breastfeeding.

Participants who experience the following events or conditions will be permanently discontinued from rIL-2:

- Toxicity requiring permanent discontinuation
- Participant or physician request to discontinue

For all toxicities that require rIL-2 to be temporarily or permanently discontinued, relevant clinical and laboratory tests will be repeated as needed until there is final resolution or stabilization of the toxicity.

2. rIL-2 schedule modifications based on virologic monitoring

Viral load determinations based on monthly intervals following rIL-2 therapy will provide the basis for comparison with individual baseline values. Since transient fluctuations in HIV-1 RNA copy number are quite common independent of cytokine therapy and may be triggered by a variety of unrelated stimulatory phenomena (e.g. intercurrent infection or other illness), single time point rises in viral load will not, by themselves, be absolute grounds for either discontinuing rIL-2 therapy or commencing antiretroviral therapy. Note that the same viral load assay should be used for making comparisons.

Assuming a maximal variability in the viral load assay of ~0.3 log and a change of at least ~0.5-0.7 log in serial samples as being truly different, a significant change in HIV-RNA from baseline is defined as ~1.0 log (0.3 log + 0.7 log). Monitoring of viral load changes in rIL-2 recipients (Groups B and C) will be as follows:

- A baseline viral load, defined as the mean of the two protocol-mandated viral loads (drawn within 45 days prior to randomization), will be established for each participant.
- Viral load determinations measured at four weeks following rIL-2 therapy will provide the basis for:
  - comparison with individual baseline values;
  - comparison with corresponding values in the other groups; and
  - comparison to determine whether rIL-2 therapy should continue.

In this regard, the following algorithm (Figure 3) will be used for withholding rIL-2 in response to sustained increases in viral load:

**Figure 3. Algorithm for withholding rIL-2 in response to sustained increases in viral load**

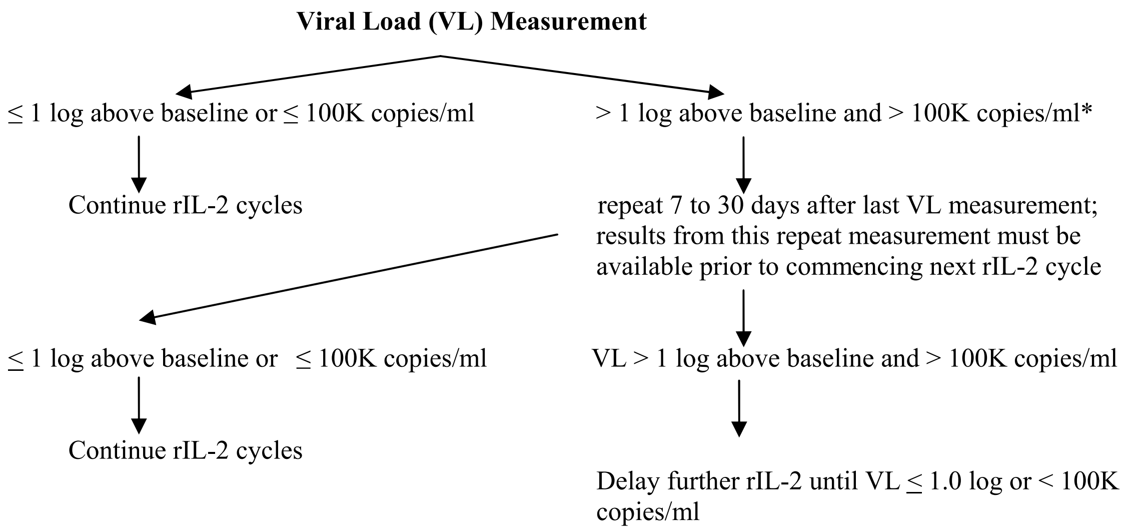


* *rIL-2 administration must stop if the criteria in these guidelines are met. Investigators may use more conservative criteria (i.e., stop rIL-2 at lower viral load levels) if it is believed to be in the best interest of the participant.*

3. rIL-2 schedule modifications based upon immunologic monitoring

$CD4^+$ T cell count determinations based on monthly intervals following rIL-2 therapy will provide the basis for comparison with individual baseline values and for determining whether rIL-2 therapy will continue. Like HIV-RNA, fluctuations in $CD4^+$ T cell count are common. Single time point decreases in $CD4^+$ T cell count will not, by themselves, be absolute grounds for discontinuing rIL-2 therapy. rIL-2 will be stopped if a participant's $CD4^+$ T cell count falls ≥25% from baseline on two consecutive measurements taken 7 to 29 days apart and at least 28 days after the start of an rIL-2 cycle (participants should be free of intercurrent illnesses or infections when these values are determined).

## D. Antiretroviral guidelines and dose modification

1. Antiretroviral dose modifications for management of toxicities

In participants randomized to the arm receiving rIL-2 in combination with HAART administered only during rIL-2 cycles, adverse events associated with antiretroviral use will also be graded according to the Toxicity Table (Appendix B) used in association with the participant's clinical presentation.

The decision to modify therapy will be based on the clinical judgment of the site investigator in discussion, as appropriate, with other investigators. Participants who do not meet objective criteria for a dose-limiting adverse event but who find the effects intolerable, may also have their antiretrovirals modified according to the schedules described below.

Recipients of antiretrovirals who develop toxicities will be managed as medically appropriate for the signs and symptoms of the toxicity. Since maximal suppression of HIV-1 replication is most likely to be accomplished by delivery of combinations of antiretroviral agents at their current recommended maximum tolerable doses, dose reduction may compromise this goal and predispose the participant to the emergence of drug-resistant viral variants. For this reason, dose reduction of antiretroviral medications will not be employed. Should a participant develop a significant complication that is believed to be the result of an antiretroviral-related toxicity, all antiretroviral medications will be stopped.

If the participant develops antiretroviral-related toxicities before the initiation of rIL-2 administration, rIL-2 administration will be withheld until a new antiretroviral regimen is restarted and tolerated for 3 days. rIL-2 administration should then commence as described in section VII.B.1. If antiretroviral-related toxicities occur during the administration of rIL-2, both antiretrovirals and rIL-2 will be stopped. This rIL-2 cycle will be considered complete and the participant will wait 8 weeks (± 2 weeks) before starting a new cycle. At that time, a new antiretroviral regimen will be attempted and, if tolerated for 3 days, rIL-2 will be administered in combination with antiretrovirals as outlined in section VII.B.

2. Antiretroviral modifications in response to rIL-2 modifications

In participants randomized to the arm receiving rIL-2 combined with HAART administered only during rIL-2 cycles, antiretroviral medications for study purposes will not be given in the absence of a concomitant rIL-2 cycle. If rIL-2 administration is withheld within a cycle for a rIL-2-related toxicity but is likely to be restarted before that cycle ends, antiretroviral therapy may be continued through the rIL-2 cycle as described in Section VII.B.1. If a rIL-2 cycle is terminated for rIL-2-related toxicities and rIL-2 dosing is unlikely to restart within that cycle, antiretrovirals should be terminated 2 days after the last rIL-2 dose.

## E. Concomitant Therapy

1. Antiretroviral therapy

Investigators will monitor participants at regular intervals to determine the needs for antiretroviral therapy in accordance with currently accepted guidelines for the management of

persons with HIV-1 infection (e.g., The U.S. Department of Health and Human Services Guidelines for the Use of Antiretroviral Agents in HIV-Infected Adults and Adolescents). Investigators may recommend that participants commence continuous antiretrovirals or participants may independently decide to commence continuous antiretrovirals for any reason. The decision to initiate antiretroviral therapy should be made upon sound clinical evidence that doing so would be in the interest of the participant, their health, and well-being. Antiretroviral therapy may be started at any time during the study, and will be managed according to good clinical practices. The rationale for change in antiretroviral therapy will be noted clearly in the case report forms.

2. Antimicrobial prophylaxis

Participants whose $CD4^+$ T cell count falls below 200 cells/$mm^3$ should commence prophylaxis for *Pneumocystis carinii* pneumonia. In participants with a reduction in $CD4^+$ T cell count to less than 100 cells/$mm^3$, prophylaxis for *Mycobacterium avium* should be initiated. Participants with a history of recurrent Herpes simplex infection who are in receipt of suppressive therapy with acyclovir or similar medication at the commencement of study therapy may continue to receive prophylaxis.

3. Treatment of intercurrent illnesses

Participants who develop intercurrent illnesses during the trial should receive appropriate systemic therapy followed by appropriate suppressive therapy thereafter. Treatment with rIL-2 may be delayed for the management of acute signs and symptoms of intercurrent illnesses. Generally the maximum interval between the first three treatment cycles should not be extended by more than 11 calendar weeks.

4. Symptomatic management during rIL-2 treatments

The use of medications to prevent or relieve symptoms during treatment with rIL-2 is encouraged. Corticosteroid administration will not be permitted during rIL-2 cycles.

## VIII. STUDY PROCEDURES

### A. General

Prior to implementation of this protocol, sites must have the protocol and consent form approved by their local regulatory authorities. These approvals must be forwarded to your regional coordinating center who will forward them to the DAIDS Regulatory Compliance Center. The DAIDS Regulatory Compliance Center will review the site-specific informed consents to ensure that they contain all the information necessary to comply with the U.S. Federal Regulations. The DAIDS Regulatory Compliance Center must approve these documents prior to the site implementing the protocol or consenting any participants.

Written informed consent must be obtained before any study-specific tests or evaluations are performed. Laboratory tests in addition to those identified in this protocol that are necessary to

assess participant safety may be performed at the discretion of the investigator. Any additional research tests should be approved by the appropriate Institutional Review Board and presented to the participant in the informed consent. Centralized testing will be used for viral genotyping only. All other tests will be performed by laboratories designated by each STALWART site. A participant should have all protocol-mandated $CD4^+$ T cell determination performed on the same flow cytometer and all HIV load determinations made by the same validated commercial assay.

**B. Baseline Screening and Enrollment**

Prior to randomization participants will attend a screening/baseline visit to document eligibility, obtain informed consent and collect baseline data for the trial.

Data collection requirements are as follows (within 45 days prior to randomization):

a. Provision of written informed consent
b. HIV-1 ELISA and confirmatory test if not already available
c. Complete medical history and clinical evaluation
d. Serum or urine pregnancy test for women of childbearing potential within 14 days prior to randomization
e. Laboratory Tests
    i. Complete blood count (CBC) with differential
    ii. Serum chemistries (electrolytes, creatinine, glucose, calcium, magnesium, AST, ALT, alkaline phosphatase, bilirubin and albumin)
    iii. Fasting (only water for at least 8 hours) serum lipids (total cholesterol, LDL,HDL and triglycerides)
    iv. TWO measures of HIV load at least 24 hours apart (any validated commercial assay)
    v. TWO measures of absolute $CD4^+$ T lymphocyte count and percent $CD4^+$ cells at least 24 hours apart
    vi. Thyroid stimulating hormone (TSH)
f. Stored plasma and serum for future virologic, immunologic, treatment-related toxicity and co-infection (e.g. viral hepatitis)-related research
g. Stored leukocyte sample for future virologic and immunologic research (e.g., human leukocyte antigen testing) and chemokine receptor testing

Participants who are randomized to receive rIL-2 should begin the first cycle within 21 days (± 7 days) of the randomization visit. For participants randomized to receive rIL-2, a pre-cycle visit must be completed and the results of all tests be known to be in keeping with study criteria before the initiation of a rIL-2 cycle.

**C. Study evaluations**

Follow up will continue until a common closing date 12 months after the last participant has been randomized. All study participants will be seen at least monthly during the first 32 weeks of the study and at least every 4 months thereafter. For participants in all three treatment groups, Hepatitis B antigen and Hepatitis C antibody will be obtained at baseline (Hepatitis C test can be eliminated if the participant has tested positive previously; both tests can be eliminated if the

participant has had negative results within the past year), and a thyroid stimulating hormone (TSH) measurement will be obtained at baseline and at week 32. In addition the participants in the two rIL-2 groups will be monitored closely during each cycle of rIL-2. See Appendix E for the Time and Events Schedule.

1. Group A Participants (no therapy)

These participants will require the same follow-up as rIL-2-treated participants, with the exception of peri-rIL-2 cycle visits. It is important that these participants are reviewed at regular intervals to ensure continuity of follow-up and to curtail premature withdrawal from the study.

2. Group B and C Participants (rIL-2 recipients)

Participants will be taught, where possible, how to self-administer rIL-2 and will also be educated about the use of prophylactic medications for the management of side effects. The decision to allow the participant to self-administer rIL-2 will be made by the investigational team.

It is intended that the pre-cycle visit for cycle 1 will coincide with week 4; pre-cycle visit for cycle 2 will coincide with week 12; and pre-cycle visit for cycle 3 will coincide with week 20.

To assess the individual participant's eligibility for rIL-2 therapy, the information listed below must be collected within 96 hours prior to the start of all cycles of rIL-2.

a. Complete blood count (CBC) with differential
b. Serum chemistries (electrolytes, creatinine, glucose, calcium, magnesium, AST, ALT, alkaline phosphatase, bilirubin and albumin)
c. Urine or plasma pregnancy test for women of childbearing potential

On day 5 (or the last day of rIL-2 administration; ± 1 day) of the first three dosing cycles the following laboratory measures will be collected:

a. Complete blood count (CBC) with differential
b. Serum chemistries (electrolytes, creatinine, glucose, calcium, magnesium, AST, ALT, alkaline phosphatase, bilirubin and albumin)
c. Stored plasma and serum for future virologic, immunologic, treatment-related toxicity and co-infection (e.g. viral hepatitis)-related research

These laboratory measurements should also be performed on day 5 (or the last day of rIL-2 administration; ± 1 day) of subsequent cycles based on experience from a participant's first three cycles and clinician discretion.

3. HIV Genotyping

In addition to the above laboratory assessments, participants in Group C will have blood drawn on cycle 3, day 5 (or the last day of rIL-2 administration, ± 1 day), for HIV resistance

genotyping. For those participant samples that demonstrate antiretroviral resistance mutations, plasma from cycle 1, day 5 (or week 4) will be sent for HIV genotyping.

**D. Follow-up Evaluations (after Week 32)**

Participants in all treatment groups will be seen every 4 months (± 2 months) after the initial 32 week study period for at least 12 months after randomization. Participants in the rIL-2 groups will also be monitored closely during each cycle of treatment. Data collection and evaluations required for 4-month follow-up visits and during each cycle of rIL-2 are described below. $CD4^+$ cell counts, HIV RNA levels and other relevant findings determined from these visits will be shared with participants.

1. 4-Month Follow-up Visits (All Groups)

   a. Targeted history and clinical evaluation
   b. Concomitant treatments, including changes in antiretroviral therapy
   c. Absolute $CD4^+$ cell count and percent $CD4^+$ cells and documentation of up to 2 interim $CD4^+$ cell counts and percents (if available)
   d. Plasma HIV RNA level (any validated commercial assay) and documentation of up to 2 interim plasma HIV RNA levels (if available)
   e. Plasma sample will be obtained from each participant for future virologic, immunologic, treatment-related toxicity and coinfection (e.g., viral hepatitis) research

2. rIL-2 Cycles (rIL-2 Groups Only)

Administration of rIL-2 therapy after the initial 32 week period should be guided on an individual basis through periodic monitoring of $CD4^+$ cell counts evaluated at least every 4 months from baseline.

As a guideline, participants will be encouraged to receive additional cycles of rIL-2 for the duration of the study to maintain their $CD4^+$ level ≥ 150 cells/mm$^3$ above baseline or ≥ 1000 cells/mm$^3$, whichever is lower. Thus, participants for whom the $CD4^+$ cell count after the third cycle of treatment is not ≥ baseline + 150 cells/mm$^3$ or ≥ 1000 cells/mm$^3$ will be encouraged to continue treatment with rIL-2 every 8 weeks until the desired $CD4^+$ cell count is attained. When participants achieve this $CD4^+$ count, their next cycle of treatment may be postponed until their $CD4^+$ count declines below this value. This flexibility and these decision guidelines apply to any cycle of therapy occurring after the first three cycles have been completed.

The following data will be collected for each cycle of treatment initiated:

   a. Toxicities resulting in dose reduction or cycle interruption
   b. Assessment of adherence to daily injections
   c. A $CD4^+$ T cell count and HIV RNA measurement within Day 28 to 34 days post cycle

Note: Women of childbearing potential must have a negative pregnancy test (urine or serum) within 96 hours prior to the start of each cycle of rIL-2 therapy as described in Section VIII.C.2.

## E. Stored Samples and Future Research

Blood will be collected and stored for future research on HIV, the immune system or related conditions. Samples will be stored with a code and will be used only by STALWART-associated investigators. In some cases, Institutional Review Board (IRB) approval may be necessary in order to use stored samples.

# IX. STUDY TREATMENT DISCONTINUATION

Participants will have their rIL-2 discontinued if any of the following events occur:

- Termination of the study
- Pregnancy
- A medically significant, grade 4 toxicity occurs that is attributable to rIL-2
- Dose-limiting toxicities at the minimal permissible rIL2 dose for this protocol (1.5 MIU)
- In Group C, intolerance to all antiretroviral regimens
- Changes in virologic or immunologic parameters occur which require a discontinuation of study therapy (see Section VII.C.)
- Malignancy requiring systemic therapy
- Participant non-compliance
- The principal investigator believes it is in the best interest of the participant
- Participants will be withdrawn from the study if they withdraw their consent to participate.

# X. STATISTICAL CONSIDERATIONS

## A. Sample Size

Sample size was determined to ensure power for each of the three planned pair-wise comparisons. The study has been powered to detect a 50 to 75 cells/mm$^3$ difference for each comparison even though the difference for some comparisons is likely to be much larger. A difference of 50 to 75 cells was chosen because it was felt differences of that size, say between groups B and C, would be important to detect and could influence how antiretroviral therapy was used with rIL-2. Thus, if a non-significant difference is found, the study is designed to be able to rule out the possibility that differences between groups are greater than 60 cells/mm$^3$. Since there are several other important outcomes besides CD4$^+$ cell count change (e.g., grade 3/4 events and changes in serum lipids), this sample size will ensure adequate power for comparisons among group for those outcomes as well.

The following additional assumptions were made:

- Power = 0.80
- Type I error = 0.05/3 (critical value = 2.41); 2-sided test

- SD of $CD4^+$ cell count change after 6 months = 135 cells/mm$^3$. This was estimated from the United Kingdom Vanguard Study from a model that included baseline $CD4^+$ cell count as a covariate.

Table 2 gives sample size estimates for three $CD4^+$ cell count differences and for two estimates of the standard deviation (SD). Based on this table, sample size was set at 160 per group. With 480 participants (160 per group), differences as small as 50 cells/mm$^3$ can be detected with 80% power even if the SD is 135 cells/mm$^3$. If the SD is 175 cells/mm$^3$, 40 cells higher than in the United Kingdom Vanguard Study, power is excellent for detecting a 60 cells/mm$^3$ difference. A higher SD may result since one group is receiving both IL-2 and HAART.

**Table 2: Sample Size Calculations**

Power = 0.80, Type I Error = 0.05
After Adjustment for 3 Pair-wise Comparisons
(Z = 2.41)
Total Sample size (all 3 groups)

| | **SD** | |
|---|---|---|
| **Hypothesized Treatment Difference (cells/mm$^3$)** | **135** | **175** |
| 50 | 465 | 780 |
| 75 | 210 | 345 |
| 100 | 120 | 195 |

## B. Data Collection and Analyses

Data will be collected on Case Report Forms and stored in a central database at the Minnesota Coordinating Center.

The NIAID Data and Safety Monitoring Board will review interim analyses for safety. These analyses will include summaries of primary and secondary outcome measures and will consider the possibility of bias resulting from the unblinded design.

Study monitors, the Food and Drug Administration and representatives of the trial sponsor, the NIAID Division of AIDS, may access study files.

The primary analysis of $CD4^+$ T cell count change after 32 weeks will be intent-to-treat using stratified analysis of variance with strata defined by participating National Trial Coordinating Center and with baseline $CD4^+$ cell count as a covariate. Baseline $CD4^+$ T cell count will be defined as the average of two pre-randomization counts obtained within 45 days prior to randomization. All three pairwise comparison will be performed using the pre-specified significance level (0.05/3 = .0167). Adjusted 95% confidence intervals will be cited. In addition, longitudinal regression models with random participant effects and area under the curve will be used to compare trends in $CD4^+$ T cell counts, viral load levels, and serum lipids over follow-up. Changes in $CD4^+$ T cell count will also be assessed at 12 months using these same analysis tools.

For all three treatment groups, participant characteristics at baseline (including age, gender, $CD4^+$ T cell count, and viral load) will be summarized by randomized treatment using appropriate summary measures.

Subgroup analyses according to baseline viral load and demographic characteristics (including prior antiretroviral treatment) will be carried out for all major response variables. These analyses will be summarized with 2df interaction tests.

For groups B and C dosage reductions of rIL-2 and grade 3 and 4 signs and symptoms will also be summarized using time to event methods. Grade 3 and 4 signs and symptoms will be reported on all randomized participants for the duration of the study.

The time to therapy modification will be summarized for each treatment arm using Kaplan-Meier plots.

For the purpose of this study, a therapy modification is defined as:

- permanent discontinuation of rIL-2 in groups B and C;
- changes in antiretroviral regimen in group C due to toxicity; and
- initiation of continuous antiretroviral therapy in any groups.

Data from monthly follow-up visits will also be used to compare treatment groups for antiretroviral therapy and opportunistic infection prophylaxes prescribed. Other descriptive analyses including the frequency distributions of the number of rIL-2 cycles received, dosage used and interruptions and dosage reductions due to toxicities will be carried out.

For Group C, participants with HIV resistance mutations detected on blood samples collected during the third rIL-2 cycle will have HIV genotyping performed on plasma collected during the first cycle. The number and types of new resistance mutations that develop between the first and third cycles will be summarized.

## XI. HAZARDS/DISCOMFORTS/RISKS

### A. Treatments

1. rIL-2

The most prominent side effects of IL-2 occur 2-6 hours following dosing and may be associated with peak plasma levels of rIL-2. Most toxicities resolve within 5 days of completion of the treatment cycle, reflecting the short half-life of the agent. Toxicities associated with rIL-2 are listed below.

The most common side effects include flu-like symptoms such as fever, chills and fatigue, nausea, rash, and redness or nodules in the areas where rIL-2 is injected. Other side effects include vomiting, diarrhea, abdominal pain, anorexia, myalgias, arthralgia, sweats, elevated hepatic transaminases, pruritis, mucositis, flushing, hyper- and hypothyroidism, elevated serum glucose, anxiety, sleeping problems, change in mood, headache, dizziness, abnormal kidney function, vascular leak and edema, hypotension, tachycardia, decreases in serum calcium, magnesium, phosphate, sodium, anemia, thrombocytopenia, shortness of breath, cough, wheezing and nasal/sinus congestion, cholecystitis. The following side effects have occurred infrequently: severe allergic reactions, angina, arrythmias, myocardial infarction or congestive heart failure , development or worsening of certain auto-immune/inflammatory diseases, neurological or psychiatric side effects such as delerium, mania, depression, attempted suicide, loss of consciousness, convulsions, memory loss, cerebral edema, optic neuritis, stroke, renal failure, worsening of viral hepatitis and liver failure, venous or arterial thromboses, pancreatitis, development or worsening of Kaposi's sarcoma, significant bacterial infections, death. rIL-2 may lead to a temporary increase in plasma HIV viremia. Some participants who have received rIL-2 and are subsequently given radiological contrast material have developed reactions including fever, chills, nausea, vomiting, pruritis, diarrhea, hypotension, swelling, and oliguria.

rIL-2 has not been studied adequately in pregnant women. Animal studies do not suggest that rIL-2 causes birth defects, but there is limited human data.

2. Antiretrovirals

Antiretroviral medications have risks that vary according to the specific drugs used and the duration for which they are used. The common side effects and risks of relevant antiretroviral medications should be reviewed with participants.

**B. Procedures**

1. Phlebotomy

The primary risks of phlebotomy include occasional bleeding or bruising of the skin at the site of needle puncture, and the sensation of transient lightheadedness with rare fainting. Blood drawing will not exceed 450 ml over any 6-week period.

2. rIL-2 Injections

rIL-2 injections can cause injection site pain, tenderness, nodules and rarely injection site ulceration and infection

### C. Stored Samples, Medical Records and Confidentiality

There is a risk that information from a research subject's medical records or results obtained from stored samples could be inadvertently released without a subject's permission.

## XII. EVENT REPORTING

In addition to the data collected at regularly scheduled follow-up visits, other important data items are reported when the site becomes aware of the event rather than waiting until the next scheduled follow-up visit. These data are described below. Reports on these data will be distributed as follows:

- Expedited Adverse Events will be sent to the NIAID Division of AIDS RCC Safety Office and the Minnesota Coordinating Center. The NIAID Division of AIDS RCC Safety Office will forward EAEs that meet the requirements of an IND Safety Report to the US Food and Drug Administration FDA, and the Minnesota Coordinating Center will forward Safety Reports to investigators for IRB/EC review and to the DSMB.

- Grade 3 and 4 Event and death reports will be sent to the Minnesota Coordinating Center. These will then be forwarded to IRBs/ECs and the DSMB for review

- Primary and Secondary Outcome Measures will be collected centrally at the Minnesota Coordinating Center and will be reviewed by the DSMB.

### A. Disease Progression Events (All treatment groups)

All progression of disease events (see Appendix D) will be reported for participants in all treatment groups for the duration of the trial irrespective of treatment status. These events should be reported immediately after a working diagnosis of the event has been made and the necessary documentation has been assembled. All events that a participant experiences must be reported, not just the first event.

### B. Grade 3 and 4 Events and Death (All treatment groups)

Grade 3 and 4 signs and symptoms (grade 3 and 4 events other than those related to an HIV disease progression diagnosis or limited to laboratory abnormalities) and deaths that occur will be reported for participants in all treatment groups for the duration of the trial irrespective of treatment status. These events should be reported immediately following site awareness. In addition, for participants in the rIL-2 groups, reasons for dosage reductions, cycle interruptions, and permanent discontinuation of treatment will be reported at the end of each cycle.

### C. Expedited Adverse Experiences (rIL-2 Groups Only)

The Division of AIDS (DAIDS), NIAID, is the sponsor for the rIL2 Investigational New Drug Application (IND) filed at the U.S. Food and Drug Administration (FDA). In accordance with the FDA Code of Federal Regulations (CFR), the IND sponsor and investigators participating in a clinical trial are responsible for the proper reporting of Expedited Adverse Experiences (EAEs). The purpose of reporting these EAEs is to better understand the toxicity and safety of investigational agents. EAE reporting and monitoring also assist in alerting the FDA, sponsor, and clinical investigators of real and potential participant safety issues. NIAID must report EAEs that are serious, unexpected, and related to the study drug to the FDA in the form of a written IND Safety Report. In general, IND Safety Reports must be submitted to the FDA as soon as possible, but no later than 15 calendar days after the sponsor is notified of the EAE. Deaths and life-threatening events with any possible relationship to a study drug must be reported to the FDA within 7 calendar days of the sponsor's awareness.

In order for NIAID to meet these timelines sites must report EAEs to their regional coordinating center within 3 working days of site awareness. These EAEs will be immediately forwarded by the regional coordinating center to the DAIDS Regulatory Compliance Center where they will be reviewed. DAIDS will ensure that the required safety information for the IND is sent to the FDA and the appropriate local authorities.

1. EAE Reporting to DAIDS

The expedited adverse event (EAE) reporting requirements and definitions for this study are defined in "The Manual for Expedited Reporting of Adverse Events to DAIDS" (*DAIDS EAE Manual*), dated May 6, 2004. AEs reported on an expedited basis must be documented on the DAIDS Expedited Adverse Event Reporting Form (*EAE Reporting Form*). The *DAIDS EAE Manual* and *EAE Reporting Form* will be supplied to each study site, will be available on the STALWART study website, and also will be available in the STALWART study procedures manual.

2. EAE Reporting Requirements

This study uses the Intensive Level of expedited AE reporting as defined in the *DAIDS EAE Manual*. The study agents that must be considered in determining relationships of AEs requiring expedited reporting to DAIDS are: Aldesleukin (rIL-2).

The Toxicity Table that must be used for grading EAEs is included in Appendix B of this protocol, will be available on the STALWART study website, and also will be available in the STALWART study procedures manual.

EAEs must be reported on an expedited basis at the Intensive Level during the entire study duration for an individual participant (from study enrollment until study completion or discontinuation of the participant from study participation for any reason). After the end of the protocol-defined EAE reporting period states above, sites must report serious, unexpected, clinical suspected adverse drug reactions if the study site staff becomes aware of the event on a passive basis, i.e., from publicly available information.

3. STALWART EAE Definition

A consideration for expedited adverse experience reporting to of AEs DAIDS is the judgment of causal association (relationship) between an AE and rIL-2. The study physician makes the site's final assessment of the causal relationship based upon the temporal relationship to administration of the rIL-2 and pharmacology of rIL-2, and his/her clinical judgment. A suspected adverse drug reaction (SADR) is an adverse event that could potentially have a causal relationship to the rIL-2 (definitely, probably, possibly, probably not related, or for deaths, pending) as defined in the *DAIDS EAE Manual*.
The following events must be reported as EAEs if they occur at any time during the study:

- Death
- Permanent disability/incapacity (except caused by an AIDS-associated diagnosis)
- Congenital anomaly or birth defect in offspring born to participants during protocol follow-up.

The following EAEs must be reported only if they occur during dosing or within 8 weeks after the most recent dose of rIL-2:

- SADR requiring hospitalization or prolongation of hospitalization
- All other grade 3 or grade 4 SADRs (with the exception of these expected events which do not have to be reported: fever, myalgia, headache and elevated thyroid stimulating hormone)

## XIII. MONITORING

ESPRIT National Coordinating Centers will monitor their respective sites for compliance to the protocol as well as ICH GCP.

The NIAID Data Safety Monitoring Board (DSMB) will review available data at least yearly. The DSMB, FDA, NIAID or other regulatory authorities can make recommendations for study continuation, modification or termination.

## XIV. BENEFITS

IL-2 monotherapy or IL-2 with peri-cycle antiretrovirals may produce increases in $CD4^+$ T lymphocyte count, but the clinical implications of these increases are not known. Study participants may benefit from frequent medical monitoring done as a part of this study.

## XV. COMPENSATION

No financial compensation will be provided to study participants other than site-specific reimbursement for expenses incurred as a consequence of participating in this study. rIL-2 and study-related routine clinical monitoring tests will be provided free of charge to all participants.

## XVI. FINANCIAL DISCLOSURE

NIH scientists who are co-investigators on this trial performed the research that discovered that IL-2 could lead to the expansion of the CD4+ T cell pool. In accordance with the Bayh-Dole technology transfer Act of 1980 these discoveries were filed with the US patent office and three US patents have been awarded to the US government in conjunction with this work. Also in accordance with this Act, these inventors are eligible to receive a portion of any royalty payments made to the US government in accordance with the licensure of these patents. At present the Chiron Corporation has an exclusive license to this technology.

## XVII. REFERENCES

1. Vittinghoffer, Scheer S, O'Malley P et al. Combination antiretroviral therapy and recent declines in AIDS incidence and mortality. *J Infect Dis* 1999. 179:717-720.

2. Mocroft A, Vella S, Benfield TL et al. Changing patterns of mortality across Europe in patients infected with HIV-1. The EuroSIDA Study Group. *Lancet* 1998. 352:1725-1730.

3. Palella FJ, Delaney, KM, Moorman AC et al. Declining morbidity and mortality among patients with advanced human immunodeficiency virus infection. HIV Outpatient Study Investigators. *N Engl J Med* 1998. 338:853-860.

4. Finzi D, Blankson J, Siliciano JD et al. Latent infection of $CD4^+$ T cells provides a mechanism for lifelong persistence of HIV-1, even in patients on effective combination therapy. *Nat Med* 1999. 5:512-517.

5. Chun TW, Carruth L, Finzi D et al. Quantification of latent tissue reservoirs and total body viral load in HIV-1 infection. *Nature* 1997. 387:183-188.

6. Wong JK, Hezareh M, Gunthard HF et al. Recovery of replication-competent HIV despite prolonged suppression of plasma viremia. *Science* 1997. 278: 1291-1295.

7. Carr A, Cooper DA. Adverse effects of antiretroviral therapy. *Lancet* 2000. 356:1423-1430.

8. Haubrich RH, Little SJ, Currier JS, et al. The value of patient-reported adherence to antiretroviral therapy in predicting virologic and immunologic response. California Collaborative Treatment Group. AIDS . 1999; 13: 1099–1107.

9. Pillay D, Taylor S, Richmann OO. Incidence and impact of resistance against approved antiretroviral drugs. *Rev Med Virol* 2000. 10:231-253.

10. Mellors JW, Munoz A, Giorgi JV, et al. Plasma viral load and $CD4^+$ lymphocytes as prognostic markers of HIV-1 infection. *Ann Intern Med* 1997. 126(12):946-54.

11. Kovacs JA, Baseler M, Dewar RJ, et al. Increases in CD4 T lymphocytes with intermittent courses of interleukin-2 in patients with human immunodeficiency virus infection. A preliminary study. *N Engl J Med 1995.* 332(9):567-75.

12. Kovacs JA, Vogel S, Albert JM, et al. Controlled trial of interleukin-2 infusions in patients infected with the human immunodeficiency virus. *N Engl J Med* 1996. 335(18):1350-58.

13. Carr A, Emery S, Lloyd A, et al. A randomised controlled, multicentre outpatient trial of intravenous or subcutaneous interleukin-2 in HIV-infected patients. *J Infect Dis* 1998. 178:992-999.

14. Davey RT, Chaitt DG, Piscitelli, et al. Subcutaneous administration of interleukin-2 in human immunodeficiency virus type 1-infected persons. *J Infect Dis* 1997.175:781-9.

15. Levy Y, Capitant C, Houhou S, et al. Comparison of subcutaneous and intravenous interleukin-2 in asymptomatic HIV-1 infection: a randomised controlled trial. ANRS 048 study group. *Lancet* 1999. 353(9168):1923-9.

16. Losso MH, Belloso WH, Emery S, et al and the Vanguard Study Group. A Randomized, Controlled, Phase II Trial Comparing Escalating Doses of Subcutaneous Interleukin-2 plus Antiretrovirals versus Antiretrovirals Alone in Human Immunodeficiency Virus Infected Patients with $CD4^+$ Cell Counts 350/mm3. *J Infect Dis* 2000. 181:1614-21.

17. Ruxrungtham K, Suwanagool S, Tavel JA, et al. A randomized, controlled 24- week study of intermittent subcutaneous interleukin-2 (IL-2) in HIV-1 infected patients in Thailand. *AIDS* 2000. 14:2509-13.

18. Hengge UR, Goos M, Esser S, et al. Randomized, controlled phase II trial of subcutaneous interleukin-2 in combination with highly active antiretroviral therapy (HAART) in HIV patients. *AIDS* 1998. 12:F225-34.

19. Larsen CS, Ostergaard L, Moller BK, Buhl MR. Subcutaneous interleukin-2 in combination with anti-retroviral therapy for treatment of HIV-1 infected subjects. *Scand J Infect Dis* 2000. 32:153-60.

20. Davey R, Murphy R, Graziano F, et al. Immunologic and virologic effects of subcutaneous interleukin 2 in combination with antiretroviral therapy: a randomized trial. *JAMA* 2000;284:183-189.

21. Davey RT, Chaitt D, Albert JM, et al. A randomized trial of high- versus low-dose subcutaneous interleukin-2 outpatient therapy for early human immunodeficiency virus type 1 infection. *J Infect Dis* 1999. 179(4):849-58.

22. Abrams DI, Bebchuk JD, Denning ET, et al Randomized, open-label study of the impact of two doses of subcutaneous recombinant interleukin-2 on viral burden in patients with HIV-1 infection and $CD4^+$ cell counts of > or = 300/mm3: CPCRA 059. *J Acquir Immune Defic Syndr* 2002 Mar 1;29(3):221-31.

23. Davey R, Chaitt DG, Kovacs JA, et al. Long-term follow-up of an early HIV-infected cohort receiving intermittent outpatient treatment with subcutaneous interleukin-2 (scIL-2). [Abstract 41230]. 12th World AIDS Conference 1998. Geneva, Switzerland.

24. Emery S, Capra W, Cooper DA, et al. Pooled analysis of 3 randomized controlled trials of interleukin-2 therapy in adult human immunodeficiency virus type 1 disease. *J Inf Dis* 2000. 182:428-34.

25. Youle M, Fisher M, Nelson M, et al. Randomised study of intermittent subcutaneous interleukin-2 (IL-2) therapy without antiretrovirals versus no treatment. [Abstract LbOr28]. XIII International AIDS Conference 2000. Durban, South Africa.

26. Chiron Corp, Proleukin® (aldesleukin) Investigator's Brochure, 2004.

# APPENDIX A

## SAMPLE INFORMED CONSENT
## for protocol:

## A Randomized, Open-Label, International Study of Subcutaneous Recombinant Interleukin-2 (rIL-2, Aldesleukin) with and without Concomitant Antiretroviral Therapy in Patients with HIV-1 infection and $CD4^+$ Cell Counts ≥300/$mm^3$

**ALL SITE INSTRUCTION THAT IS INCLUDED IN A TEXT BOX SHOULD BE REMOVED FROM THE SITE'S INFORMED CONSENT FOR PARTICIPANTS**

OHRP Requirements to be read by the sites:

PLEASE NOTE THAT THIS SAMPLE LANGUAGE DOES NOT PREEMPT OR REPLACE LOCAL EC/IRB REVIEW AND APPROVAL. INVESTIGATORS ARE REQUIRED TO PROVIDE THE LOCAL EC/IRB WITH A COPY OF THIS SAMPLE LANGUAGE ALONG WITH THE LANGUAGE INTENDED FOR LOCAL USE. LOCAL IRBS ARE REQUIRED TO WEIGH THE UNIQUE RISKS, CONSTRAINTS, AND POPULATION CONSIDERATIONS AS A CONDITION OF ANY APPROVAL. ANY DELETION OR SUBSTANTIVE CHANGE OF INFORMATION CONCERNING RISKS OR ALTERNATIVE TREATMENT MUST BE JUSTIFIED BY THE INVESTIGATOR, APPROVED BY THE LOCAL EC/IRB, AND NOTED IN THE EC/IRB MINUTES. JUSTIFICATION AND EC/IRB APPROVAL OF SUCH CHANGES MUST BE FORWARDED TO THE REGIONAL COORDINATING CENTER FOR SUBMISSION TO THE DIVISION OF AIDS (DAIDS) . SPONSOR-APPROVED CHANGES IN A DAIDS PROTOCOL MUST BE APPROVED BY THE LOCAL EC/IRB BEFORE USE UNLESS INTENDED FOR THE ELIMINATION OF APPARENT IMMEDIATE HAZARD. NEW INFORMATION SHALL BE SHARED WITH EXISTING PARTICIPANTS AT NEXT ENCOUNTER, WITH ALL NEW PARTICIPANTS PRIOR TO INVOLVEMENT, OR AS THE LOCAL EC/IRB MAY OTHERWISE ADDITIONALLY REQUIRE.

**TITLE OF CLINICAL TRIAL:**
Study of Aldesleukin with and without Antiretroviral Therapy (ESPRIT 002: STALWART) (Version 1.0)

**PRINCIPAL INVESTIGATOR:** **PHONE:**

## INTRODUCTION

You are being asked to take part in the research study named above because you are infected with HIV, the virus that causes AIDS, and because you have a CD4 cell (also called T4 helper cells) count of at least 300 cells/$mm^3$.

This is a study of an investigational drug called interleukin-2 (IL-2 or Aldesleukin). Before you can decide whether or not to take part in this study, we would like to explain the purpose of the study, how it may help you, any risks to you, and what is expected of you.

## YOUR PARTICIPATION IS VOLUNTARY

This consent form gives you information about the study that will be discussed with you. Once you understand the study, and if you agree to take part, you will be asked to sign this consent form. You will be given a copy to keep.

Before you learn about the study, it is important that you know the following:

- Your participation is entirely voluntary;
- You may decide not to take part or to withdraw from the study at any time without losing the benefits of your routine medical care.

**PURPOSE OF STUDY**

IL-2 is produced naturally in the body and helps CD4 cells multiply. CD4 cells help fight infection. People with HIV infection have low levels of natural IL-2. Although IL-2 is not approved for treatment of HIV by the United States Food and Drug Administration, it has been studied in human trials and most (but not all) participants who have received IL-2 in combination with anti-HIV drugs show increases in their $CD4^+$ cell count. One study showed that, over a six month period, most (but not all) of 24 participants who were given IL-2 without anti-HIV drugs had an increase in CD4 cell counts without increasing HIV viral load compared to 12 participants who received no therapy. Although these results are encouraging, comparison with other studies suggests that giving IL-2 in combination with anti-HIV drugs may produce a greater increase in $CD4^+$ cell count compared to administering IL-2 alone. We are conducting this study:

1. to determine the safety and tolerance of IL-2 taken alone (without anti-HIV drugs);
2. to determine the safety and tolerance of IL-2 when administered with anti-HIV drugs given during IL-2 cycles only;
3. to see if the use of IL-2 alone or with anti-HIV drugs taken around IL-2 cycles causes an increase in CD4 cell count greater than no therapy at all;
4. to see if the use of IL-2 alone or with anti-HIV drugs taken around IL-2 cycles causes changes in HIV viral load that are different compared to no therapy at all.

This study will enroll about 480 people around the world. The study will last about 2 years.

**PROCEDURES**

**Study Treatment**

If you qualify for this study, you will be assigned by chance (like by the roll of dice) to one of three treatment groups. You will have an equal (1 to 1 to 1) chance of being assigned to one of the three groups. The groups are:

1. Standard of care treatment (Group A): This group will not receive IL-2 or anti-HIV drugs, although individuals may begin anti-HIV drugs at any time they or their doctors believe it would be in their best interest to do so. Anti-HIV drugs will not be supplied to you through this study. This group is important to determine whether or not IL-2 as used in the other two groups may be helpful or harmful to the immune system or HIV load in the blood.

2. IL-2 alone group (Group B): This group will receive IL-2 alone. IL-2 will be given at a dose of 7.5 million international units (MIU) by injection below the skin, twice a day, for 5 days in a row (called a "cycle" of IL-2). This will be followed by 7 weeks when participants do not get IL-2. Another 5-day cycle of getting IL-2 by injection will follow after the 7 weeks of not getting IL-2. This routine will be repeated a third time. Then individuals in this group may continue to get IL-2 by injection for 5 days in a row, starting no closer than 6 weeks apart, if the study doctor determines that IL-2 may be helping them. If you are assigned to receive IL-2, your doctor or nurse will talk with you about how you will receive your IL-2 injections. If possible, you will be taught how to prepare and give yourself the injections of IL-2. Individuals in this group may begin anti-HIV drugs at any time they or their doctors believe it would be in their best interest to do so. Anti-HIV drugs will not be supplied to you through this study.
3. IL-2 plus anti-HIV drugs around IL-2 cycles only (Group C): This group will receive three initial cycles of IL-2 as well as additional cycles if the study doctor determines that IL-2 may be helping them as outlined for Group B above but will also receive 10 days of anti-HIV therapy around IL-2 cycles (3 days before, 5 days during and 2 days after IL-2 cycles). If you are assigned to this group, your doctor will discuss with you what anti-HIV therapy you will receive. Individuals in this group may begin continuous treatment with anti-HIV drugs at any time they or their doctors believe it would be in their best interest to do so. Anti-HIV drugs will not be supplied to you through this study. In this group, IL-2 must be administered along with anti-HIV drugs. So if you must permanently stop anti-HIV drugs, further cycles of IL-2 will not be administered.

**During the Study**

You will have at least one clinic visit to see if you are eligible to participate in the study before you can be randomized to one of the three study groups. This visit must occur within 45 days prior to randomization. At this visit about 30 to 50 milliliters (6 to 10 teaspoons) of blood will be drawn. Some of the blood will be used to measure the amount of HIV in your blood and your CD4 cell count. Some of the blood tests done at this visit will require that you have not had anything to eat and only water to drink for at least 8 hours prior to the visit. We will also check to see if you are infected with certain viruses that infect the liver (Hepatitis B and C). These results will be shared with you at your next study visit or when they become available.

Whether you are assigned to receive IL-2 or not, you will return to the clinic at least every 4 weeks for at least 7 additional visits. At these visits you will have a physical exam and about 30 to 50 milliliters (6 to 10 teaspoons) of blood will be drawn. Some of the blood will be used to measure the amount of HIV in your blood and your CD4 cell count. These results will be shared with you at your next study visit or when they become available. The blood tests done at the seventh visit will require that you have not had anything to eat and only water to drink for at least 8 hours prior to the visit.

After the first 7 visits of the study, you will be asked to come to the clinic at least every 4 months for a total duration of up to 2 years. At these visits you may see your study doctor or nurse and will have about 30 to 50 milliliters (6 to 10 teaspoons) of blood drawn. Some of the blood will

be used to measure the amount of HIV in your blood and your $CD4^+$ cell count. These results will be shared with you on or before your next 4-month visit.

If you are assigned to receive IL-2, you will be required to return to the clinic for additional visits on the fifth day of the injections. At these visits you will be asked to confirm that you were able to take all your IL-2 injections, and you will have about 30 to 50 milliliters (6 to 10 teaspoons) of blood drawn. If you develop side effects from IL-2, your doctor may want to see you more often. Additional blood or urine samples may be needed to determine how the rIL-2 is affecting you. Your doctor may also decrease your dose of IL-2 because of side effects. Women of childbearing potential will have a pregnancy test within 4 days prior to the start of each cycle of rIL-2.

If you were randomized to receive IL-2 treatments and complete 3 cycles of IL-2, individualized recommendations about the frequency and the dose of future IL-2 treatments will be made. These recommendations will be based upon your response to IL-2 therapy as measured by your $CD4^+$ cell count. The overall goal of IL-2 therapy is to administer IL-2 at the minimum dose and frequency that is required to maintain your $CD4^+$ cell count at 150 cells/mm$^3$ above baseline or >1000 cells/mm$^3$, whichever value is lower. You may continue to receive IL-2 for a maximum of 5 days and every 8 weeks for up to two years. The dose of IL-2 will not exceed 7.5 MIU twice daily and will not be less than 1.5 MIU twice daily.

To receive cycles of IL-2, your doctor will see you for a clinical exam and/or blood tests to ensure that you are well before you receive each cycle. You will then be seen on the last day of the dosing cycle and once again about one month after each cycle. You may need to be seen even more frequently if you have a history of serious side effects during prior IL-2 treatments, or if you develop such side effects with future treatments.

Some of the blood drawn from you as part of this study will be used for genetic tests. These tests will be done in order to provide information that is important to HIV research. As part of this HIV research, these tests will provide information about the immune system. This information may help researchers better understand the immune system's effect on the course of HIV. Genetic tests can also tell researchers about diseases passed on to you by your parents or from you to your children. The results of these genetic tests will be used for research purposes only. Neither you nor your doctor will be given the results from these tests.

In addition, some blood will be stored and used for future studies of the HIV virus, the immune system and complications of IL-2 therapy and anti-HIV drugs.

To protect your privacy, a code number will be used to identify all your blood samples. This code number will make it hard for any information to be traced to you, but there is still some risk to your privacy. Only STALWART-approved investigators will be able to use your coded blood samples for any future tests.

While you are taking part in this study, you may be asked to take part in some smaller related research studies. You may refuse to take part in these smaller studies and still be in this main study.

You should tell your nurse or doctor before you take any other medications or enroll in other clinical trials.

**RISKS and/or DISCOMFORTS**

**Risks of IL-2**
The IL-2 used in this study may have side effects, some of which are listed below. Please note that these lists may not include all the possible side effects that could be seen with IL-2. These lists include the more serious or common side effects with a known or possible relationship. If you have questions concerning additional side effects, please ask the medical staff at your site.

Potential Risks with IL-2

The most common side effects include:
- Flu-like symptoms such as fever, chills, feeling tired
- Nausea
- Rash
- Redness or hard lumps in the areas where IL-2 is injected; the lumps may take weeks to months to resolve

Other side effects include:
- Vomiting, loose or watery stools, stomach pain, loss of appetite
- Joint or muscle aches, sweats
- Abnormal liver tests, which may mean liver damage
- Skin peeling or itching
- Sores in the mouth
- Flushing
- Abnormal function of the thyroid gland
- High blood sugar
- Anxiety, sleeping problems, change in mood, headache, dizziness
- Abnormal kidney function
- Swelling, which is caused by leakage of fluid from the blood vessels into the surrounding tissues
- Low blood pressure and fast heartbeat
- Decreases in the amount of certain elements in the blood (calcium, magnesium, phosphate, sodium)
- A decrease in the number of red blood cells, which may cause weakness, dizziness, and fatigue
- A decrease in the number of platelets, which help the blood to clot
- Shortness of breath, cough, wheezing and nasal/sinus stuffiness
- Inflammation of the gallbladder

The following side effects have occurred infrequently:

- Severe allergic reactions, with breathing/swallowing difficulties and hives
- Serious heart problems, which may include angina (chest pain), abnormal heart rhythm, heart attacks or decreased pumping ability
- Development or worsening of certain auto-immune/inflammatory diseases
- Serious neurological or psychiatric side effects (for example, delerium, mania, depression, attempted suicide, loss of consciousness, convulsions, memory loss, swelling of the brain, or nerve disorders, such as disease of the nerve supplying the eye)
- Damage to an area of the brain or to other organs, caused by low blood supply
- Kidney failure (which may be fatal)
- Worsening of viral hepatitis and liver failure
- Blood clot formation in a vein or artery
- Inflammation of the pancreas
- Development or worsening of Kaposi's sarcoma
- Significant bacterial infections
- Death

There is a risk of serious and/or life threatening side effects when other medications are taken with IL-2. It is possible that IL-2 may increase the risk of side effects of certain drugs.

Sometimes, when people who have received IL-2 are given a certain type of dye for X-ray examinations, a reaction may occur that includes symptoms such as fever, chills, nausea, vomiting, itchiness rash, diarrhea, low blood pressure, swelling, and low urine output.

IL-2 has not been studied adequately in pregnant women. Animal studies do not suggest that IL-2 causes birth defects, but it is not known whether or not this is true in humans.

**Risks of Anti-HIV Drugs**

The antiretrovirals used in this study may have side effects. Although some of these side effects are seen only after someone has taken anti-HIV drugs for extended periods of time, others may occur after short periods of use. If you are randomized to receive anti-HIV drugs, your study doctor or nurse will discuss the possible side effects of these drugs.

**Risks of not taking anti-HIV drugs**

Studies have proven that participants with a high HIV load develop infections and may die sooner than do participants with lower viral loads. Studies have also suggested that strong anti-HIV drug combinations that lower the levels of virus in the body delay the onset of AIDS or death. Guidelines recommend that anti-HIV drug combinations of at preferably three or more drugs be prescribed for participants with symptoms of HIV infection, low CD4 counts, or other conditions indicating progressive HIV disease. It remains controversial as to what precise level of CD4 count these guidelines should be implemented for participants. However, while not every physician necessarily agrees with all of these guidelines, very few doubt that, properly used, these drugs can bring about a reduction in viral load in many participants. Participants who decide not to take anti-HIV drugs for whatever reason may therefore be depriving themselves of the potential benefits of these drugs in lowering the viral load and possibly slowing down the progression of AIDS.

**Risks of taking IL-2 without anti-HIV drugs**
IL-2 is known to activate T cells, and HIV can more readily replicate in activated T cells. Thus there is a risk that IL-2 can lead to an increase in HIV in the blood or organs. In earlier studies of IL-2 in participants with CD4 counts greater than 200 cells/$mm^3$, a temporary increase in the viral load in the blood was seen at the end of IL-2 therapy in some participants, despite the fact that those participants were also taking anti-HIV drugs. The viral load returned to baseline in most participants by the one-month follow-up visit. But without anti-HIV drugs it is possible that the viral load will not return to its baseline level by the one month follow-up visit or any point thereafter. The long-term effects of such a persistent rise in viral load upon the course of HIV infection are not fully understood, but it could lead to more rapid progression of HIV disease. While we will not allow your viral load to remain elevated above a safety range for a long time without recommending that anti-HIV drugs be started, it is possible that damage to your immune system could be occurring during this time. It is possible that anti-HIV drugs may be less effective in treating HIV if IL-2 has raised your viral load than if it had been started earlier when your viral load was lower. The results of this could be that you will gain less benefit from anti-HIV drugs.

There is also the possibility that increases in viral load caused by IL-2 could reduce or eliminate the favorable effect of IL-2 therapy upon the CD4 count. If this occurs, it could undermine the main purpose of undergoing IL-2 therapy. It is also possible that your CD4 count could fall to below its baseline value, in which case having taken no treatment might have been better than participating in this protocol.

**Risks of taking anti-HIV drugs intermittently**
The primary risk of taking anti-HIV drugs intermittently is the risk of developing resistance to anti-HIV drugs. When HIV becomes resistant to anti-HIV drugs, the virus is no longer suppressed by these medications. Also, other similar anti-HIV drugs may be ineffective at suppressing virus that becomes drug resistant.

Many studies that interrupt anti-HIV drugs do so only once participants have achieved a controlled, low-level of HIV in their blood. In this study, you may be assigned to start and stop anti-HIV drugs before you achieve such a low level of HIV. We do not know if using anti-HIV drugs in this manner can increase your risk of developing HIV that is resistant to medications.

**Risk of Transmitting Disease**
The risk of giving an injection by needle at home is the small chance of a needle stick injury to non-infected people in your household, which can cause an infectious disease (such as HIV, HBV, and/or HCV). Clinic personnel will review with you what to do if there is an accidental needle stick. Careful disposal of used needles in special containers that will be provided should be practiced.

**Risks of Blood Drawing**
The risks of having blood taken include discomfort, bleeding, bruising, lightheadedness, fainting and rarely infection or a blood clot where the needle enters the body.

## PREGNANCY AND BREASTFEEDING

Pregnant women cannot join the study since we do not know whether IL-2 is safe for them or their unborn babies. If you are a woman who is able to become pregnant, you must have a negative pregnancy test before you join this study. If you join the study and are assigned to get IL-2, you must also have a negative pregnancy test prior to each five-day period of IL-2 injections. If you become pregnant during the study, you should tell your study doctor or nurse right away. If you become pregnant your IL-2 will be stopped. However, you will be asked to continue your follow-up visits. Women who become pregnant may be able to start taking IL-2 again after their pregnancy is over.

Women who are breastfeeding cannot join the study since we do not know whether IL-2 may pass through breast milk and may harm the baby. Taking IL-2 does not guarantee that you will decrease the risk of passing HIV through your breast milk to the baby. If you decide to breastfeed your baby your IL-2 will be stopped. However, you may be able to start IL-2 again after you stop breastfeeding.

## BENEFITS

We do not know whether being in this study will benefit you. If you join the study and get IL-2, it may raise your CD4 cell count. However, even if it does, we do not know whether this increase will improve your health. What we learn from this study may help us to improve the treatment of other people who are infected with HIV.

## FUTURE AVAILABILITY OF IL-2

After you complete this trial, neither the sponsor of this trial nor the company that produces IL-2 will provide IL-2 to you since we do not know if IL-2 as used in this study will improve your health.

## NEW FINDINGS

You will be told of any new information learned during the study that might cause you to change your mind about staying in the study. At the end of the study, you will be told when study results may be available and how to learn about them.

## WITHDRAWING FROM THE STUDY

If you enroll in this study, you may decide to withdraw at any time, but we would like you to come for one final visit to see how you are doing and to draw blood to evaluate your $CD4^+$ T cell count and HIV viral load.

## REASONS WHY IL-2 MAY BE STOPPED WITHOUT YOUR CONSENT

You may be taken off IL-2 temporarily or permanently without your consent if:

- Your CD4 cell count consistently falls below its baseline

- Your HIV viral load consistently rises above its baseline
- You become pregnant or begin breastfeeding
- You have an illness that the doctor feels would make it too difficult to tell what effect IL-2 was having on you
- You develop cancer or other disease that requires you to take chemotherapy
- You have a side effect from IL-2 that requires temporary discontinuation of IL-2
- You are randomized to Group C but cannot tolerate any anti-HIV drugs
- The investigator decides that continuing to take IL-2 would harm you
- You need a treatment not allowed on this study
- The study is cancelled

**ALTERNATIVES TO PARTICIPATION**

Alternatives to participating in this study include:
- Not taking any treatment for your HIV infection;
- Taking approved anti-HIV drugs prescribed by your doctor;
- Taking investigational drugs available from your doctor or another research study.

**COSTS TO YOU**

During the study, if you are in the group that gets IL-2, we will give it to you for free. You, your insurance company, or some other third-party payer must pay for other drugs including anti-HIV drugs and drugs needed to prevent or treat infections related to HIV. We will provide all clinical and professional services, diagnostics, and lab work that are part of this study and not part of your regular care at no cost to you.

*Site Instruction:*
*If the above information is not correct for your country/site, please revise the text to explain to the subject any costs to them that may result from participating in the study and obtaining their anti-HIV drugs.*

**CONFIDENTIALITY**

Your research records will be confidential to the extent permitted by law. You will be identified by a code, and personal information from your records will not be released without your written permission. You will not be personally identified in any publication about this study. However, your records may be reviewed by the United States agency that regulates the research (the Food and Drug Administration); the United States agency sponsoring the research (the National Institute of Allergy and Infectious Diseases); study monitors; study personnel, members of local and national ethics committees or Institutional Review Boards, and the pharmaceutical company that is supplying the IL-2 (Chiron Corporation).

*Site Instruction:*
*If there are any other country/site-specific organizations or personnel that might have access to your participants' research records, please add them to the above text.*

**RESEARCH-RELATED INJURY**

If you are injured because of being in this study, the [*insert the name of the clinic*] will give you immediate necessary treatment for your injuries. The cost for this treatment will be charged to you or your insurance company. You will then be told where you may receive additional treatment for injuries. There is no program for monetary compensation or other forms of compensation for such injuries. You do not give up any of your legal rights by signing this form.

*Site Instruction:*
*If the information is not correct for your country/site, please revise the information to inform the subject of the following: 1. what treatment will be provided to the participants, 2. who will pay for the treatment, 3. if there is any plan for compensation for research-related injury issues, such as lost wages, etc. If you specify that your country/site will not provide compensation, you must also state that NIAID has no plan to compensate participants.*

*Site Instruction:*
*If there are any country/site specific regulations or guidance on financial disclosures, please add appropriate language to the informed consent document. Sample language is provided below:*

**FINANCIAL DISCLOSURE**

Scientists at the U.S. National Institutes of Health (NIH) who are co-investigators on this trial performed the research that discovered that IL-2 could lead to an increase in CD4+ cell numbers. The U.S. government holds the patent (exclusive rights) for the use of IL-2 in HIV-infected patients, and these investigators and NIH may benefit financially for this discovery. By law, investigators are eligible to receive payments for their discoveries. It is also possible that new patents and/or technologies may result from the results of the STALWART trial that could lead to financial benefit by the NIH investigators. Please discuss with your study doctor any questions you may have about these issues.

**PROBLEMS OR QUESTIONS**

If you ever have questions about this study or in case of research-related injuries, you should contact [*insert the name of the study doctor at your site]* at [*insert the telephone number*], or if you have questions about research subject's rights you can call [*insert the name and title of the appropriate country/site-specific person]* at [*insert the telephone number*].

*Site Instruction:*
*U.S. regulations do not require, but recommend, that the person to contact about research subject rights be someone not directly involved with the research.*

**SIGNATURE PAGE**

*Site Instruction:*
This is only a suggested Signature Page. Sites may use their own Signature Page.

If you have read and had the informed consent document explained to you, and if you understand the information and you voluntarily agree to join this study, please sign your name below.

| | | |
|---|---|---|
| ______________ | ____________________ | ____________________ |
| Volunteer's Name (typed or printed) | Volunteer's Signature | Date |
| OR | OR | |
| ____________________ | ____________________ | ____________________ |
| Volunteer's Legal Guardian or Representative | Legal Guardian's Signature | Date |
| ______________ | ____________________ | ____________________ |
| Witness' Name (Typed or printed) | Witness' Signature | Date |

**NOTE: This consent form with the original signatures MUST be retained on file by the principal investigator. A copy must be given to the volunteer. A copy should be placed in the volunteer's medical record, if applicable.**

**A witness to the subject's signature is strongly encouraged.**

**APPENDIX B**
**TOXICITY TABLE FOR GRADING ADVERSE EVENTS**

The following table will be used for grading events which occur during the study, regardless of treatment assignment or study drug status. All events should be assessed for their level of seriousness, and this assessment documented in the patient's record.

## Estimating severity grade

For abnormalities NOT found elsewhere on the Toxicity Table use the scale below to estimate grade of toxicity:

GRADE 1 Transient or mild discomfort; no limitation in activity; no medical intervention/therapy required.

GRADE 2 Mild to moderate limitation in activity - some assistance may be needed; no or minimal medical intervention/therapy required.

GRADE 3 Marked limitation in activity, some assistance usually required; medical intervention/therapy required, hospitalization possible.

GRADE 4 Extreme limitation in activity, significant assistance required; significant medical intervention/therapy required, hospitalization or hospice care.

| HEMATOLOGY | GRADE 1 | GRADE 2 | GRADE 3 | GRADE 4 |
|---|---|---|---|---|
| Hemoglobin | 8.0 - 9.4 g/dL **OR**<br>80 - 94 g/L **OR**<br>4.93 - 5.83 mmol/L | 7.0 - 7.9 g/dL **OR**<br>70-79 g/L **OR**<br>4.31 - 4.92 mmol/L | 6.5 - 6.9 g/dL **OR**<br>65 - 69 g/L **OR**<br>4.03 - 4.30 mmol/L | <6.5 g/dL **OR**<br><65 g/L **OR**<br><4.03 mmol/L |
| Absolute Neutrophil Count | 1000 -1500/$mm^3$ **OR**<br>1.0 - 1.5/G/L* | 750 - 999/$mm^3$ **OR**<br>0.75 -0.99/G/L* | 500 - 749/$mm^3$ **OR**<br>0.5 - 0.749/G/L* | <500/$mm^3$ **OR**<br><0.5/G/L* |
| Platelets | 75000 - 99000/$mm^3$<br>**OR** 75 - 99/G/L* | 50000 - 74999/$mm^3$<br>**OR** 50 - 74.9/G/L* | 20000 - 49999/$mm^3$<br>**OR** 20 - 49.9/G/L* | <20000/$mm^3$<br>**OR** <20/G/L* |
| Prothrombin Time (PT) | >1.0 - 1.25 X ULN | >1.25 - 1.5 X ULN | >1.5 - 3.0 X ULN | >3.0 X ULN |
| PTT | >1.0 - 1.66 X ULN | >1.66 - 2.33 X ULN | >2.33 - 3.0 X ULN | >3.0 X ULN |
| Methemoglobin | 5.0 - 10.0% | 10.1 - 15.0% | 15.1 - 20.0% | >20% |
| *G = X $10^9$ | | | | |

| CHEMISTRIES | GRADE 1 | GRADE 2 | GRADE 3 | GRADE 4 |
|---|---|---|---|---|
| SODIUM | | | | |
| Hyponatremia | 130 - 135 meq/L **OR** 130 - 135 mmol/L | 123 - 129 meq/L **OR** 123 - 129 mmol/L | 116 - 122 meq/L **OR** 116 - 122 mmol/L | <116 meq/L **OR** <116 mmol/L |
| Hypernatremia | 146 - 150 meq/L **OR** 146 - 150 mmol/L | 151 - 157 meq/L **OR** 151 - 157 mmol/L | 158 - 165 meq/L **OR** 158 - 165 mmol/L | >165 meq/L **OR** >165 mmol/L |
| POTASSIUM | | | | |
| Hyperkalemia | 5.6 - 6.0 meq/L **OR** 5.6 - 6.0 mmol/L | 6.1 - 6.5 meq/L **OR** 6.1 - 6.5 mmol/L | 6.6 - 7.0 meq/L **OR** 6.6 - 7.0 mmol/L | >7.0 meq/L **OR** >7.0 mmol/L |
| Hypokalemia | 3.0 - 3.4 meq/L **OR** 3.0 - 3.4 mmol/L | 2.5 - 2.9 meq/L **OR** 2.5 - 2.9 mmol/L | 2.0 - 2.4 meq/L **OR** 2.0 - 2.4 mmol/L | <2.0 meq/L **OR** <2.0 mmol/L |
| PHOSPHATE | | | | |
| Hypophospha-temia | 2.0 - 2.4 mg/dL **OR** 0.63 - 0.77 mmol/L | 1.5 - 1.9 mg/dL **OR** 0.46 - 0.62 mmol/L | 1.0 - 1.4 mg/dL **OR** 0.32 - 0.45 mmol/L | <1.0 mg/dL **OR** <0.32 mmol/L |
| CALCIUM - (corrected for albumin) | | | | |
| Hypocalcemia | 7.8 - 8.4 mg/dL **OR** 1.95 - 2.10 mmol/L | 7.0 - 7.7 mg/dL **OR** 1.74 - 1.94 mmol/L | 6.1 - 6.9 mg/dL **OR** 1.53 - 1.73 mmol/L | <6.1 mg/dL **OR** <1.53 mmol/L |
| Hypercalcemia | 10.6 - 11.5 mg/dL **OR** 2.65 - 2.87 mmol/L | 11.6 - 12.5 mg/dL **OR** 2.88 - 3.14 mmol/L | 12.6 - 13.5 mg/dL **OR** 3.15 - 3.37 mmol/L | >13.5 mg/dL **OR** >3.37 mmol/L |
| MAGNESIUM | | | | |
| Hypomagnesemia | 1.2 - 1.4 meq/L **OR** 0.60 - 0.70 mmol/L | 0.9 - 1.1 meq/L **OR** 0.45 - 0.59 mmol/L | 0.6 - 0.8 meq/L **OR** 0.30 - 0.44 mmol/L | <0.6 meq/L **OR** <0.30 mmol/L |
| BILIRUBIN | | | | |
| Hyperbilirubin-emia | >1.0 - 1.5 X ULN | >1.5 - 2.5 X ULN | >2.5 - 5 X ULN | >5 X ULN |
| GLUCOSE | | | | |
| Hypoglycemia | 55 - 64 mg/dL **OR** 3.01 - 3.55 mmol/L | 40 - 54 mg/dL **OR** 2.19 - 3.00 mmol/L | 30 - 39 mg/dL **OR** 1.67 - 2.18 mmol/L | <30 mg/dL **OR** <1.67 mmol/L |
| Hyperglycemia (nonfasting and no prior diabetes) | 116 - 160 mg/dL **OR** 6.44 - 8.90 mmol/L | 161 - 250 mg/dL **OR** 8.91 - 13.88 mmol/L | 251 - 500 mg/dL **OR** 13.89 - 27.76 mmol/L | >500 mg/dL **OR** >27.76 mmol/L |
| TRIGLYCERIDES | --------------------- | 400 - 750 mg/dL **OR** 4.52 - 8.47 mmol/L | 751 - 1200 mg/dL **OR** 8.48 - 13.55 mmol/L | >1200 mg/dL **OR** >13.55 mmol/L |
| CREATININE | >1.0 - 1.5 X ULN | >1.5 - 3.0 X ULN | >3.0 - 6.0 X ULN | >6.0 X ULN |
| URIC ACID | | | | |
| Hyperuricemia | 7.5 - 10.0 mg/dL **OR** 446 - 595 umol/l | 10.1 - 12.0 mg/dL **OR** 596 - 716 umol/l | 12.1 - 15.0 mg/dL **OR** 717 - 892 umol/l | >15.0 mg/dL **OR** >892 umol/l |

| **CHEMISTRIES (continued)** | **GRADE 1** | **GRADE 2** | **GRADE 3** | **GRADE 4** |
|---|---|---|---|---|
| LIVER TRANSAMIN-ASE (LFTs) | | | | |
| AST (SGOT) | 1.25 - 2.5 X ULN | >2.5 - 5.0 X ULN | >5.0 - 10.0 X ULN | >10.0 X ULN |
| ALT (SGPT) | 1.25 - 2.5 X ULN | >2.5 - 5.0 X ULN | >5.0 - 10.0 X ULN | >10.0 X ULN |
| GGT | 1.25 - 2.5 X ULN | >2.5 - 5.0 X ULN | >5.0 - 10.0 X ULN | >10.0 X ULN |
| Alkaline Phosphatase | 1.25 - 2.5 X ULN | >2.5 - 5.0 X ULN | >5.0 - 10.0 X ULN | >10.0 X ULN |
| PANCREATIC ENZYMES | | | | |
| Amylase | >1.0 - 1.5 X ULN | >1.5 - 2.0 X ULN | >2.0 - 5.0 X ULN | >5.0 X ULN |
| Pancreatic amylase | >1.0 - 1.5 X ULN | >1.5 - 2.0 X ULN | >2.0 - 5.0 X ULN | >5.0 X ULN |
| Lipase | >1.0 - 1.5 X ULN | >1.5 - 2.0 X ULN | >2.0 - 5.0 X ULN | >5.0 X ULN |

| **CARDIO-VASCULAR** | **GRADE 1** | **GRADE 2** | **GRADE 3** | **GRADE 4** |
|---|---|---|---|---|
| CARDIAC ARRHYTHMIA | ------------------ | Asymptomatic; transient dysrhythmia, no Rx req | Recurrent/persistent dysrhythmia; symptomatic Rx req | Unstable dysrhythmia, hospitalization and Rx req |
| HYPER-TENSION | Transient, increase >20 mmHg; no Rx | Recurrent; chronic increase >20 mmHg, Rx required | Acute Rx req; outpatient hospitalization possible | Hospitalization required |
| HYPO-TENSION | Transient orthostatic hypotension, no Rx | Symptoms correctable with oral fluid Rx | IV fluid required, no hospitalization required | Hospitalization required |
| PERICARDITIS | Minimal effusion | Mild/mod asymptomatic effusion, no Rx | Symptomatic effusion, pain, EKG changes | Tamponade **OR** pericardiocen-tesis **OR** surgery required |
| HEMOR-RHAGE, BLOOD LOSS | --------------------- | Mildly symptomatic, no Rx required | Gross blood loss **OR** 1-2 units transfused | Massive blood loss **OR** >2 units transfused |

| GASTRO-INTESTINAL | GRADE 1 | GRADE 2 | GRADE 3 | GRADE 4 |
|---|---|---|---|---|
| NAUSEA | Mild **OR** transient; reasonable intake maintained | Mod discomfort **OR** intake decreased for <3 days | Severe discomfort **OR** minimal intake for ≥3 days | Hospitalization required |
| VOMITING | Mild **OR** transient; 2-3 episodes per day **OR** mild vomiting lasting <1 week | Mod **OR** persistent; 4-5 episodes per day **OR** vomiting lasting ≥1 week | Severe vomiting of all food/fluids in 24 hrs **OR** orthostatic hypotension **OR** IV Rx required | Hypotensive shock **OR** hospitalization for IV Rx required |
| DIARRHEA | Mild **OR** transient; 3-4 loose stools per day **OR** mild diarrhea lasting <1 week | Mod **OR** persistent; 5-7 loose stools per day **OR** diarrhea lasting ≥1 week | Bloody diarrhea **OR** orthostatic hypotension **OR** >7 loose stools/day **OR** IV Rx required | Hypotensive shock **OR** hospitalization required |
| ORAL DISCOMFORT/ DSYPHAGIA | Mild discomfort, no difficulty swallowing | Difficulty swallowing but able to eat and drink | Unable to swallow solids | Unable to drink fluids; IV fluids required |
| CONSTIPATION | Mild | Moderate | Severe | Distention with vomiting |

| NEUROLOGIC | GRADE 1 | GRADE 2 | GRADE 3 | GRADE 4 |
|---|---|---|---|---|
| NEURO-CEREBELLAR | Slight incoordination **OR** dysdiadochokin-esia | Intention tremor **OR** dysmetria **OR** slurred speech **OR** nystagmus | Locomotor ataxia | Incapacitated |
| NEURO-PSYCH/MOOD | -------------------- | -------------------- | Severe mood changes requiring medical intervention | Acute psychosis requiring hospitalization |
| PERIPHERAL NEUROPATHY | *Peripheral neuropathy is a syndrome characterized by signs or symptoms of one or several specific neurologic manifestations: neuromuscular paresis, paresthesia, neuromotor, and neurosensory abnormalities.* | *Peripheral neuropathy should be graded as the maximum grade, according to the DAIDS toxicity table, of any of these manifestations which, in your clinical judgment, are due to peripheral neuropathy rather than another (i.e., central) cause.* | | |

| **NEUROLOGIC (continued)** | **GRADE 1** | **GRADE 2** | **GRADE 3** | **GRADE 4** |
|---|---|---|---|---|
| NEURO-MUSCULAR PARESIS | Subjective weakness; no objective symptoms/signs | Mild objective signs, symptoms, no decrease in function | Objective weakness; function limited | Paralysis |
| NEURO-MOTOR | Decrease in reflexes **OR** patients with chronic stable abnormality of reflex or use of muscle | Absence of a previously present reflex | Absence of 2-3 previously present reflexes | Absence of >3 previously present reflexes |
| NEURO-SENSORY | Decrease in sensation (pinprick, vibratory or hot/cold) **OR** patients with chronic **STABLE** abnormality of sensation | Absence of a previously present sensory finding (one dermatome) | Absence of 2-3 previously present sensory dermatomes | Absence of >3 previously present sensory dermatomes |

| **RESPIRATORY** | **GRADE 1** | **GRADE 2** | **GRADE 3** | **GRADE 4** |
|---|---|---|---|---|
| BRONCHO-SPASM, Acute | Transient; no Rx; FEV1 <80% - 70% (or peak flow) | Rx required; normalizes with bronchodilator; FEV1 50% - <70% (or peak flow) | No normalization with bronchodilator; FEV1 25% - <50% (or peak flow), retractions | Cyanosis; FEV1 <25% (or peak flow) **OR** intubated |
| DYSPNEA | Dyspnea on exertion | Dyspnea with normal activity | Dyspnea at rest | Dyspnea requiring $O_2$ therapy |

| **URINALYSIS** | **GRADE 1** | **GRADE 2** | **GRADE 3** | **GRADE 4** |
|---|---|---|---|---|
| PROTEINURIA | | | | |
| Spot urine | 1+ | 2 - 3+ | 4+ | Nephrotic syndrome |
| 24 hour urine | 200 mg-1 g loss/day **OR** <0.3% **OR** <3 g/l | 1 - 2 g loss/day **OR** 0.3 - 1.0% **OR** 3 - 10 g/l | 2 - 3.5 g loss/day **OR** >1.0% **OR** >10 g/l | Nephrotic syndrome **OR** >3.5 g loss/day |
| GROSS HEMATURIA | Microscopic only | Gross, no clots | Gross plus clots | Obstructive **OR** transfusion req |

| MISCEL-LANEOUS | GRADE 1 | GRADE 2 | GRADE 3 | GRADE 4 |
|---|---|---|---|---|
| FEVER (oral, >12 hours) | 37.7 - 38.5$^0$C **OR** 100.0 - 101.5$^0$F | 38.6 - 39.5$^0$C **OR** 101.6 - 102.9$^0$F | 39.6 - 40.5$^0$C **OR** 103 - 105$^0$F | >40.5$^0$C **OR** >105$^0$F for ≥ 12 continuous hours |
| HEADACHE | Mild; no Rx required | Moderate **OR** non-narcotic analgesia Rx | Severe **OR** responds to initial narcotic Rx | Intractable **OR** requiring repeated narcotic Rx |
| ALLERGIC REACTION | Pruritus without rash | Localized urticaria | Generalized urticaria angioedema | Anaphylaxis |
| CUTANEOUS/RASH/ DERMATITIS | Erythema, pruritus | Diffuse maculopapular rash **OR** dry desquamation | Vesiculation **OR** moist desquamation **OR** ulceration | **ANY ONE**: mucous membrane involvement, suspected Stevens-Johnson (TEN), erythema multiforme, necrosis req surgery, exfoliative dermatitis |
| LOCAL REACTION (2% parenteral Rx - not vaccination or skin test) | Erythema | Induration <10 mm **OR** inflammation **OR** phlebitis | Induration >10 mm **OR** ulceration | Necrosis of skin |
| FATIGUE | Normal activity reduced <25% | Normal activity reduced 25-50% | Normal activity reduced >50%; cannot work | Unable to care for self |

# APPENDIX C

## GUIDELINES FOR IL-2 DOSE MODIFICATIONS

The table below outlines common IL-2 related toxicities which may require IL-2 dose modification (see Section III.A.3. Guidelines for IL-2 Dosage Modification). A cycle in which such a toxicity occurs will be interrupted until the toxicity is considered, in the opinion of the treating physician, to no longer be dose-limiting.

The guidelines below are minimum recommendations and the decision to stop, dose-modify and/or restart study drug should be based on the clinical judgement of the treating physician in discussion, as appropriate, with their NTCC, RCC and other ESPRIT investigators.

| **HEMATOLOGY** | **INTERRUPT IL-2 FOR:** | **CONSIDER RESTARTING IL-2 WHEN:** |
|---|---|---|
| Hemoglobin | ≤ 7.9 g/dL OR<br>≤ 79 g/L OR<br>≤ 4.9 mmol/L | ≥ 9.4 g/dL OR<br>≥ 94 g/L OR<br>≥ 5.8 mmol/L |
| Platelets | ≤ 50,000/mm$^3$ OR<br>≤ 50 /G/L* | > 50,000/mm$^3$ OR<br>> 50 /G/L* |
| Prothrombin Time (PT) | ≥ 1.5 X ULN | ≤ 1.2 X ULN |
| PTT | ≥ 2.3 X ULN | ≤ 1.7 X ULN |
| *G = X $10^9$ | | |

| **CHEMISTRIES** | **INTERRUPT IL-2 FOR:** | **CONSIDER RESTARTING IL-2 WHEN:** |
|---|---|---|
| SODIUM | | |
| Hyponatremia | ≤ 129 meq/L **OR**<br>≤ 129 mmol/L | ≥ 130 meq/L **OR**<br>≥ 130 mmol/L |
| POTASSIUM | | |
| Hypokalemia | ≤ 2.5 meq/L **OR**<br>≤ 2.5 mmol/L | ≥ 3.4 meq/L **OR**<br>≥ 3.4 mmol/L |
| CALCIUM - (corrected for albumin) | | |
| Hypocalcemia | < 7.0 mg/dL **OR**<br>< 1.7 mmol/L | ≥ 7.5 mg/dL **OR**<br>≥ 1.9 mmol/L |
| MAGNESIUM | | |
| Hypomagnesemia | ≤ 0.8 meq/L **OR**<br>≤ 0.4 mmol/L | ≥ 1.1 meq/L **OR**<br>≥ 0.6 mmol/L |
| HEPATIC TRANSAMINASES | > 5-10 x ULN, or any increase from baseline that physician feels to be significant. | Return of LFT's to < 2 x ULN, or to patients baseline. |
| CREATININE | ≥ 1.5 X ULN | ≤ 1.1 X ULN, or to patients baseline. |

| CARDIOVASCULAR | INTERRUPT IL-2 FOR: | CONSIDER RESTARTING IL-2 WHEN: |
|---|---|---|
| CARDIAC ARRHYTHMIA | Any dysrhythmia other than mild sinus tachycardia | Resolution of dysrhythmia |
| HYPOTENSION | Hypotension which does not resolve with IV fluids | Return to patient's baseline blood pressure |

| GASTROINTESTINAL | INTERRUPT IL-2 FOR: | CONSIDER RESTARTING IL-2 WHEN: |
|---|---|---|
| NAUSEA | Severe discomfort **OR** minimal intake for $\geq$ 2 days | Mild **OR** transient; reasonable intake maintained |
| VOMITING | Severe vomiting **OR** IV treatment required | Mild **OR** transient |
| ORAL DISCOMFORT/ DSYPHAGIA | Severe discomfort with swallowing solids | Difficulty swallowing but able to eat and drink |

| NEUROLOGIC | INTERRUPT IL-2 FOR: | CONSIDER RESTARTING IL-2 WHEN: |
|---|---|---|
| HEADACHE | Headache requiring treatment with narcotics | Substantial improvement of symptoms |
| NEURO-PSYCHIATRIC | Severe mood changes requiring medical intervention | Substantial improvement of symptoms |

| RESPIRATORY | INTERRUPT IL-2 FOR: | CONSIDER RESTARTING IL-2 WHEN: |
|---|---|---|
| BRONCHOSPASM, Acute | Treatment required; FEV1 or peak flow <70% of baseline | No Rx required; FEV1 or peak flow >80% of baseline |
| DYSPNEA | Dyspnea on exertion | No dyspnea |

| MISCELLANEOUS | INTERRUPT IL-2 FOR: | CONSIDER RESTARTING IL-2 WHEN: |
|---|---|---|
| FEVER (oral) | $\geq 39^0$C $\geq$ 6 continuous hours despite appropriate antipyretic therapy | $\leq 39^0$C or $\geq 39^0$C intermittently only |
| CUTANEOUS/RASH/ DERMATITIS | Vesiculation **OR** moist desquamation **OR** ulceration | Erythema and pruritis only |

# APPENDIX D
# AIDS-DEFINING ILLNESSES AND OTHER CONDITIONS CONSIDERED EVIDENCE OF DISEASE PROGRESSION

## CDC Category C 1993 Definition

- Candidiasis of bronchi, trachea, or lungs
- Candidiasis, esophageal
- Cervical cancer, invasive
- Coccidioidomycosis, disseminated or extrapulmonary
- Cryptococcosis, extrapulmonary
- Cryptosporidiosis, chronic intestinal (> 1 month's duration)
- CMV disease (other than liver, spleen, or nodes)
- CMV retinitis (with loss of vision)
- Encephalopathy, HIV-related (including AIDS Dementia Complex)
- *Herpes simplex*, chronic ulcers (> 1 month's duration); or bronchitis, pneumonitis, or esophagitis
- Histoplasmosis, disseminated or extrapulmonary
- Isosporiasis, chronic intestinal (> 1 month's duration)
- Kaposi's sarcoma (mucocutaneous or visceral)
- Lymphoma , Burkitt's (or equivalent term)
- Lymphoma, primary, of brain
- *Mycobacterium avium* complex or *M. kansasii*, disseminated or extrapulmonary
- *M. tuberculosis*, any site (pulmonary or extrapulmonary)
- *Mycobacterium*, other species or unidentified species, disseminated or extrapulmonary
- *Pneumocystis carinii* pneumonia
- Pneumonia, recurrent bacterial (2 documented episodes within 1 year of each other following randomization)
- Progressive multifocal leukoencephalopathy
- *Salmonella* septicemia, recurrent (2 documented episodes within 1 year of each other following randomization)
- Toxoplasmosis of brain
- Wasting syndrome due to HIV

## Additions to CDC Definition

- Aspergillosis, invasive
- Bartonellosis
- Chagas disease (American trypanosomiasis) of the CNS
- *Herpes zoster*, multi-dermatomal (≥10 lesions in a non-contiguous site)
- Leishmaniasis, visceral (kala-azar)
- Lymphoma, Hodgkin's
- Lymphoma, non-Hodgkin's, all cell types
- Microsporidiosis (> 1 month's duration)
- Nocardiosis
- *Penicillium marneffii*, disseminated
- Pneumocystis carinii, extrapulmonary
- Rhodococcus equi disease

# APPENDIX E
# TIME AND EVENTS SCHEDULE

This schedule applies to all participants (Groups A, B, and C)

| Study Requirements | Study Visits | | | | | | | | Follow-up Visits |
|---|---|---|---|---|---|---|---|---|---|
| | Baseline | Week 4 Visit[4] | Week 8 Visit[4] | Week 12 Visit[4] | Week 16 Visit[4] | Week 20 Visit[4] | Week 24 Visit[5] | Week 32 Visit[4] | Every 4 Months |
| Clinical Evaluation | X | X | X | X | X | X | X | X | X |
| Medical History | X | | | | | | | | X |
| CBC with differential | X | | | | | | | X | |
| Serum Chemistries (electrolytes, creatinine, glucose, Ca, Mg, AST, ALT, alkaline phosphatase, albumin, and bilirubin) | X | | | | | | | X | |
| Fasting Lipids (total cholesterol, LDL, HDL, triglycerides) | X | | | | | | | X | |
| Pregnancy Test[1] | X | | | | | | | | |
| Absolute $CD4^+$ Cell Count and Percent | X[2] | X | X | X | X | X | X | X | X[3] |
| HIV RNA | X[2] | X | X | X | X | X | X | X | X[3] |
| Plasma Storage (draw 10mL) | X | X | | X | | X | | X | X |
| Serum Storage (draw 10mL) | X | X | | X | | X | | X | X |
| Leukocyte Storage (from plasma tube) | X | | | | | | | | |
| Thyroid Stimulating Hormone | X | | | | | | | X | |
| HBV Ag and HCV Ab[6] | X | | | | | | | | |

[1] Urine or serum, for women of childbearing potential within 14 days of randomization

[2] Two $CD4^+$ lymphocyte counts/percents and HIV RNA determinations ≥ 24 hours apart obtained within 45 days prior to randomization, at least one of which meets eligibility criterion

[3] Including up to two interim measurements

[4] Visit window: ± 2 weeks

[5] Visit window: + 6 weeks, - 2 weeks

[6] Hepatitis C test can be eliminated if the participant has previously tested positive; both tests can be eliminated if the participant has had negative results within the past year

This additional schedule applies to IL-2 cycles in Groups B and C only.

| | **Cycle 1** | | **Cycle 2** | | **Cycle 3** | | **Additional Cycles[1]** | |
|---|---|---|---|---|---|---|---|---|
| | **PRE[2]** | **POST[3]** | **PRE[2]** | **POST[3]** | **PRE[2]** | **POST[3]** | **PRE[2]** | **POST** |
| Toxicities/Adverse Events | | X | | X | | X | | X |
| Assessment of adherence to daily injections | | X | | X | | X | | X |
| CBC with differential | X | X | X | X | X | X | X | |
| Serum Chemistries (electrolytes, creatinine, glucose, Ca, Mg, AST, ALT, alkaline phosphatase, albumin) | X | X | X | X | X | X | X | |
| Pregnancy Test[4] | X | | X | | X | | X | |
| HIV RNA and CD4+ T cell count[5] | | | | | | | | X |
| Stored plasma and serum[6] (draw 10mL for each) | | X | | X | | X | | |
| HIV Genotyping[7] (draw 10mL) | | | | | | X | | |

[1] See criteria for additional cycles Section VIII. D.2.
[2] Obtained within 96 hours prior to rIL-2 administration
[3] Obtained on day 5 (or the last day of rIL-2 administration; ± 1 day) of rIL-2 administration
[4] Urine or serum for women of childbearing potential, obtained within 96 hours prior to rIL-2 administration
[5] Obtained at day 28 to 34 post-rIL-2 cycle
[6] Obtained on day 5 (or the last day of rIL-2 administration; ± 1 day) of rIL-2 administration
[7] Group C only

**Timeline of Events**

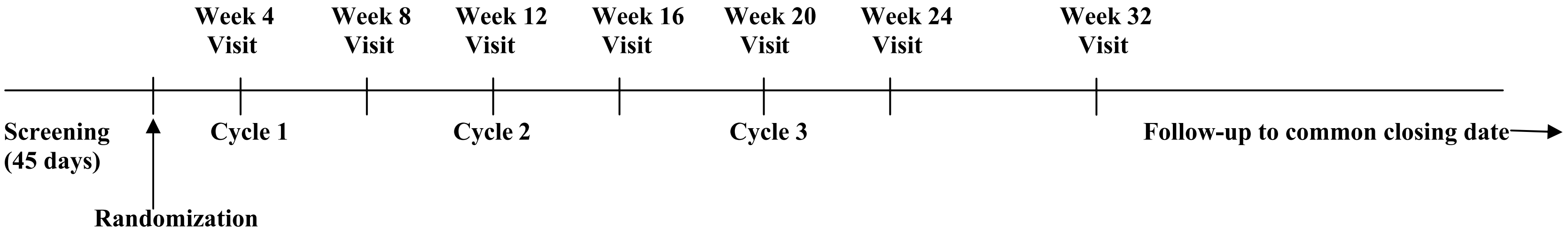

Supplement: Protocol S1 — Trial Protocol. (0.44 MB PDF) [file pone.0009334.s001.pdf]
